# CAV1 - GLUT3 signaling is important for cellular energy and can be targeted by Atorvastatin in Non-Small Cell Lung Cancer

**DOI:** 10.7150/thno.35805

**Published:** 2019-08-14

**Authors:** Azhar Ali, Elena Levantini, Chee Wai Fhu, Jun Ting Teo, John G. Clohessy, Julian L. Goggi, Chan-shuo Wu, Leilei Chen, Tan Min Chin, Daniel G Tenen

**Affiliations:** 1 Cancer Science Institute Singapore, National University of Singapore, MD6, #12-01, 14 Medical Drive, Singapore 117599.; 2 Harvard Stem Cell Institute, Harvard Medical School, Boston, MA 02215, USA; 3 Beth Israel Deaconess Medical Center, Boston, MA 02215, USA.; 4 Institute of Biomedical Technologies, National Research Council (CNR), Pisa, Italy; 5 Singapore Bioimaging Consortium (A*STAR), Helios, 02-02, 11 Biopolis Way, 138667 Singapore; 6 Gleneagles Hospital, 6A Napier Road, Singapore 258500; 7 Preclinical Murine Pharmacogenetics Facility, Beth Israel Deaconess Cancer Center, Boston, MA 02115, USA

**Keywords:** Statin, Cav1, glucose uptake, GLUT3, EGFR-TKI resistance, non-small cell lung cancer

## Abstract

**Background**: The development of molecular targeted therapies, such as EGFR-TKIs, has positively impacted the management of EGFR mutated NSCLC. However, patients with innate and acquired resistance to EGFR-TKIs still face limited effective therapeutic options. Statins are the most frequently prescribed anti-cholesterol agents and have been reported to inhibit the progression of various malignancies, including in lung. However, the mechanism by which statin exerts its anti-cancer effects is unclear. This study is designed to investigate the anti-proliferative effects and identify the mechanism-of-action of statins in NSCLC.

**Methods**: In this study, the anti-tumoral properties of Atorvastatin were investigated in NSCLC utilizing cell culture system and *in vivo* models.

**Results**: We demonstrate a link between elevated cellular cholesterol and TKI-resistance in NSCLC, which is independent of EGFR mutation status. Atorvastatin suppresses growth by inhibiting Cav1 expression in tumors in cell culture system and in *in vivo* models. Subsequent interrogations demonstrate an oncogenic physical interaction between Cav1 and GLUT3, and glucose uptake found distinctly in TKI-resistant NSCLC and this may be due to changes in the physical properties of Cav1 favoring GLUT3 binding in which significantly stronger Cav1 and GLUT3 physical interactions were observed in TKI-resistant than in TKI-sensitive NSCLC cells. Further, the differential effects of atorvastatin observed between EGFR-TKI resistant and sensitive cells suggest that EGFR mutation status may influence its actions.

**Conclusions**: This study reveals the inhibition of oncogenic role of Cav1 in GLUT3-mediated glucose uptake by statins and highlights its potential impact to overcome NSCLC with EGFR-TKI resistance.

## Introduction

Cholesterol is recognized to be important for cellular growth in mammalian cells. Apart from being a crucial constituent of cell membrane, cholesterol is an intrinsic component for cellular platforms such as lipid rafts. Lipid rafts are involved in various processes including cellular signal transduction, membrane trafficking and cholesterol transport 1. Among the proteins associated with lipid rafts, Caveolin-1 (Cav1) is an important constituent of non-planar lipid rafts, known as caveolae, and plays an important role in regulating cellular signal transduction 2, 3.

Cav1 has important roles in cancer, in which its expression has been linked to tumor initiation as well as enhanced metastatic potential, suggesting a major oncogenic function for Cav1 and implying that it may act as predictor for cancer progression 4 - 7. However, results have been reported, in which Cav1 re-expression in breast and colon cancer cells reduced the frequency of tumor formation in *in vivo* and culture systems 8, 9. In mice, *CAV1* gene disruption demonstrates phenotypic characteristics associated with type II diabetes, pulmonary defects, and increased susceptibility in developing breast cancer 10, 11. Collectively, these observations strongly indicate that Cav1 may act as a tumor suppressor or oncogene depending on the cell type in which its function is dysregulated. While the mechanism/s underlying these dissimilar phenomena remain unknown, recent lines of evidence have shown that Cav1 regulates cellular energy metabolism favoring survival 12, 13.

The development of tyrosine kinase inhibitors (TKIs), targeting the epidermal growth factor receptor (EGFR), and the sequential detection of activating EGFR mutations as a molecular marker for tumor sensitivity to these drugs, has positively impacted lung cancer management. However, non-small cell lung cancer (NSCLC) patients with innate and acquired resistance to EGFR-TKIs, face limited effective therapeutic options. Thus, a need to identify therapeutic targets that will benefit EGFR-TKI resistant patients is greatly warranted.

Statins are one of the most commonly prescribed drugs used in cardiovascular-related diseases 14, 15. Apart from lowering plasma cholesterol, statins are shown to exert other benefits including neuro-protection, reduced vascular inflammation and enhanced endothelial function 16 - 18. In addition, the use of statin is reported to offer protective effects by reducing lung cancer risk 19, 20, and is associated with improved survival of patients with Stage IV disease of both adenocarcinoma and squamous cell carcinoma subtypes 21, 22. Statins are found to benefit lung cancer patients receiving EGFR-TKI therapy with improved response rates, longer progression-free survival and overall survival 20, 23. More importantly, the combination of statins with EGFR-TKIs is shown to overcome EGFR-TKI resistance in NSCLC cells with EGFR T790M or KRAS mutations 24, 25. However, the underlying mechanism by which statins exert its anti-tumor effects in EGFR-TKI resistant NSCLC remains unclear, and it is the focus of the present study. Using cell culture systems and *in vivo* models of lung cancer, we demonstrate how the FDA-approved anti-cholesterol drug atorvastatin (ATV) disrupts cellular energy homeostasis through Cav1-GLUT3 mediated glucose uptake and restricts growth of TKI-resistant NSCLC. Given the limited therapeutic options, this study highlights the potential use of statins in the management of TKI-resistant NSCLC.

## Results

### Cholesterol is upregulated and may play a role in NSCLC

**Figure 1:**
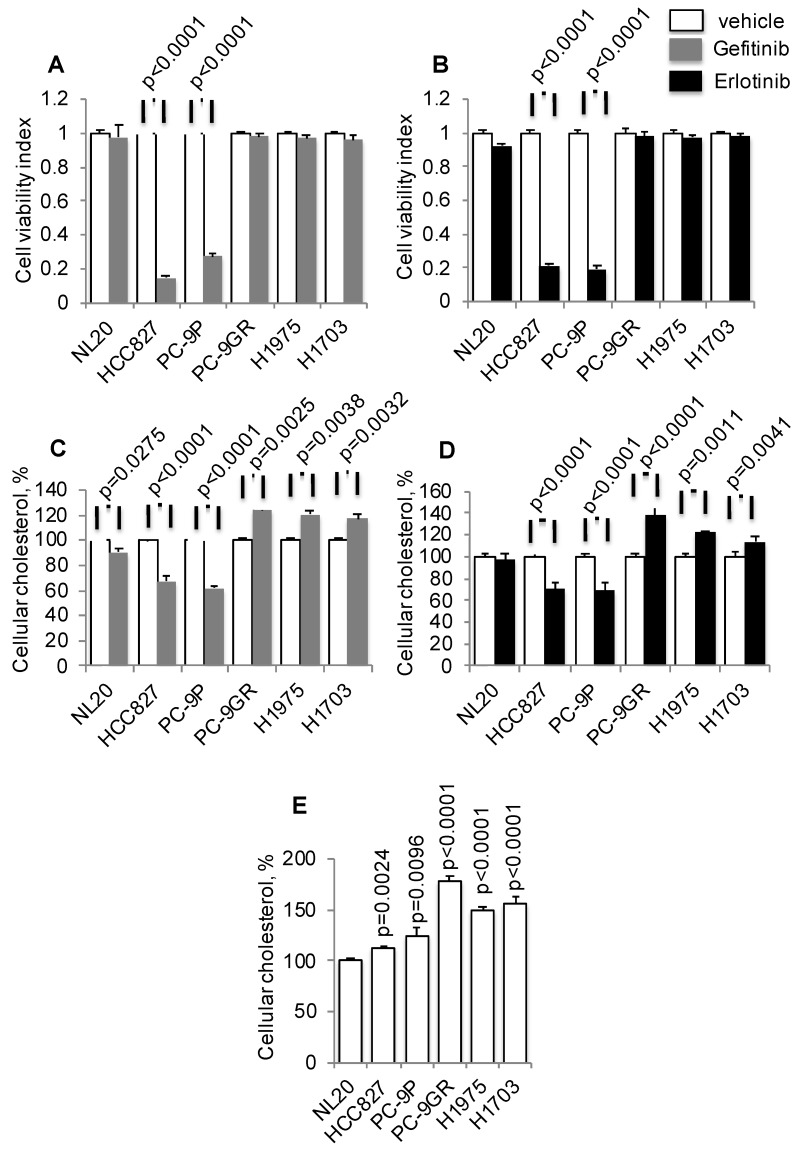
Elevated cellular cholesterol is associated with EGFR-TKI resistance in NSCLC cells. **A and B.** Cell viability assays show the effects of Gefitinib (n=3) or Erlotinib (n=3) at 1 μM on NSCLC growth after 72 h incubation. **C. and D.** Total cellular cholesterol was quantified in cells treated with vehicle or Gefitinib (1 μM, n=3) or Erlotinib (1 μM, n=3) for 72 h. Cellular cholesterol is presented as percentages and normalized to vehicle**. E.** Measurement of total cellular cholesterol (n=3) in lung cells, in which was normalized for cell number. Significance in differences in cellular cholesterol, in which vehicle acted as control, was determined by t test.

To investigate if there is a link between cholesterol levels and TKI-resistance, TKI-sensitive (PC-9 and HCC827) and -resistant (PC-9GR, H1975, and H1703) cells were incubated with Gefitinib or Erlotinib, followed by total cellular cholesterol assays. All cholesterol assays were normalized to cell number. The mutation status of EGFR in these cells is shown in Table S1. Tumor cells were exposed to a clinical dose of 1 µM Gefitinib or Erlotinib for 72 h to validate drug response, as shown in Figures 1A and 1B, respectively 26, 27. Results from cholesterol assays demonstrated that Gefitinib or Erlotinib exposure significantly led to elevated cellular cholesterol in TKI-resistant NSCLC cells compared to vehicle, as shown in Figures 1C and 1D, respectively. Exposure of cells to these TKIs, however, reduced cellular cholesterol in TKI-sensitive PC-9 cells. In immortalized non-transformed NL20 cells, included as control, the drug had a lesser effect on cholesterol inhibition compared to TKI-resistant cells. Subsequently, measurement of baseline cellular cholesterol showed that NSCLC cells had significantly higher cholesterol than NL20 cells (Figure 1E). Importantly, cellular cholesterol levels were significantly higher in TKI-resistant (PC-9GR, H1975 and H1703) than in TKI-sensitive (PC-9 and HCC827) groups (t-test, p=0.035). Further, TKI-resistant cells were resistant to EGFR silencing by siRNAs as shown in cell viability assays (Figure S1A), and cellular cholesterol levels were significantly elevated after EGFR knockdown, when compared to TKI-sensitive PC-9P cells (Figure S1B and S1C). The result showed that elevation of cholesterol levels seen in TKI-resistant cells were a consequence of TKI-resistance, therefore eliminating any potential Gefitinib/Erlotinib off-target effects. Together, these results demonstrate that cholesterol level is upregulated and may play a role in NSCLC.

### Atorvastatin exhibits anti-tumor activity and inhibits Cav1 expression in NSCLC

**Figure 2:**
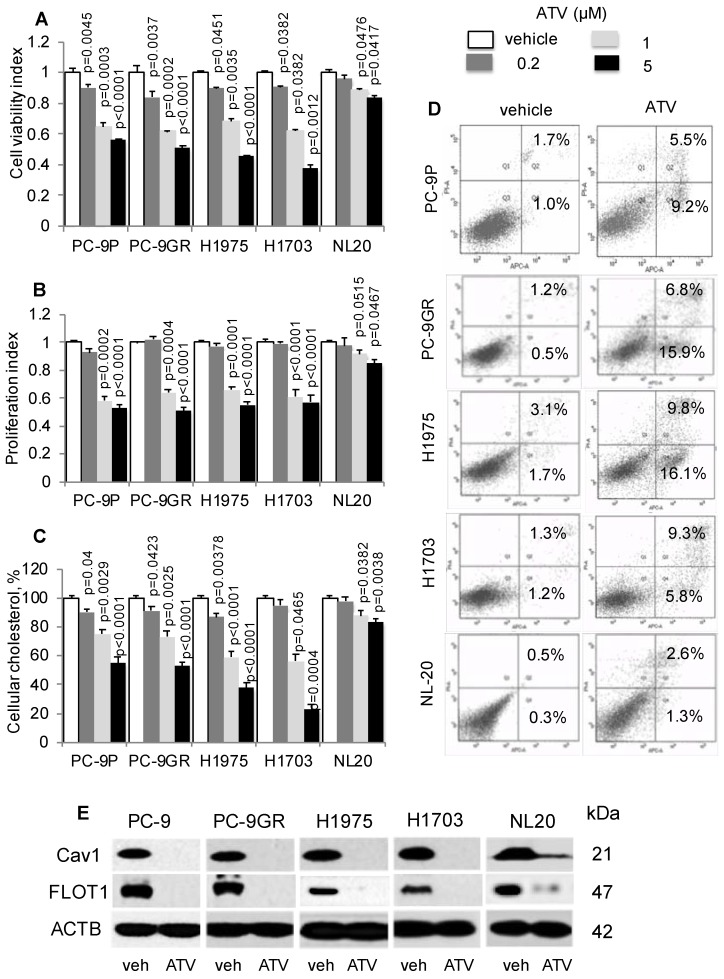
Atorvastatin (ATV) restricts NSCLC growth, and correlates with lower cellular cholesterol, induced apoptosis and downregulated CAV1 expression. **A.** Cell viability (n=3) and **B.** proliferation (n=3) assays demonstrate dose response of tumor cells treated with vehicle and atorvastatin for 72 h at the indicated concentrations. Significance in differences in viability indexes, in which vehicle acted as control, was determined by t test. **C.** Total cellular cholesterol measurement (n=3) in tumor cells treated for 72 h with vehicle or ATV at the indicated doses. Cellular cholesterol is presented as percentages and normalized to vehicle. Significance in differences in cellular cholesterol, in which vehicle acted as control, was determined by t test. **D.** Annexin V / PI staining demonstrates percentages of early (lower right quadrant) and late (upper right quadrant) apoptotic cells exposed to vehicle or ATV (at 1 μM) for 48 h (n=3). **E.** Western blot analysis of Caveolin 1 (Cav1) protein expression from cells treated with vehicle and ATV (at 1 μM) for 72 h. Protein size is indicated in kDa.

First, we determined if cholesterol is important in lung tumor cells by exposing PC-9P, PC-9GR, H1975 and H1703 NSCLC and NL20 cells to the anti-cholesterol drug atorvastatin (ATV). Cells were treated with vehicle or ATV (at 0.2, 1 or 5 μM) for 72 h. The clinical dose of ATV is 1 μM 28, and different concentrations of ATV used in this study are used to demonstrate its efficacy in killing NSCLC cells. As shown in Figure 2A, ATV strongly reduced tumor cell viability in a dose dependent manner compared to vehicle, whereas the reduction of cell viability in NL20 cells was less significant. Results from cell proliferation assays, in Figure 2B, demonstrated a similar dose-dependent downward trend, whereby after 1 μM of ATV exposure, tumor cell proliferation was strongly reduced in PC-9P, PC-9GR, H1975 and H1703 cells by 32.7% ± 4.1% (p=0.017), 35.1% ± 3.9% (p = 0.012), 29.5% ± 3.5% (p = 0.015) and 36.5% ± 4.0% (p = 0.011) respectively, compared to vehicle. Likewise, inhibition of proliferation of ATV-treated NL20 (p=0.515) cells was not as effective as in NSCLC cells (PC-9P, p = 0.002; PC-9GR, p=0.004; H1975, p<0.001; H1703, p<0.001). The importance of cholesterol for NSCLC growth was evident in ATV-treated NSCLC cells, which showed significantly reduced cholesterol, in a dose dependent manner, compared to vehicle (Figure 2C). In contrast, the decline in total cholesterol levels in ATV-treated NL20 cells was less significant. Overall, these data demonstrate that ATV significantly inhibits growth of tumor cells but is less effective in non-transformed cells at the clinically relevant dose of 1 μM.

Next, we sought to investigate the effects of ATV on cellular proliferation and cholesterol levels in Gefitinib exposed EGFR-TKI resistant cells. From Figure S2A, cell proliferation assays showed that ATV significantly reduced growth of Gefitinib-treated TKI-resistant PC-9GR (p<0.0001) and H1975 (p<0.0001) cells when compared to ATV treatment alone. In contrast, proliferation indices between Gefitinib and Gefitinib with ATV combination were similar in TKI-sensitive PC-9P (p=0.4871). Additionally, the combination treatment of ATV and Gefitinib exhibited similar anti-proliferation activity to that of ATV treatment alone in H1703 (p=0.0719) and NL20 (p=0.2283) cells (Figure S2A). Cellular cholesterol quantitation assays showed that the ATV and Gefitinib combination significantly reduced cholesterol levels in TKI-resistant PC-9GR, p=0.0093 and H1975, p=0.0049 cells when compared to ATV treatment alone (Figure S2B). Further, cholesterol levels in ATV and Gefitinib exposed H1703 (p=0.1525) and NL20 (0.4542) cells were similar to that of ATV treated cells only. In EGFR-TKI sensitive PC-9P cells, cholesterol levels between ATV alone and ATV with Gefitinib showed no difference (p=0.7242). Collectively, the data demonstrate that the combination treatment of ATV and Gefitinib demonstrate superior tumor growth suppression and concomitant reduced cellular cholesterol levels, over ATV or Gefitinib alone, in induced (PC-9GR and H1975) but not in native EGFR-TKI resistant (H1703) NSCLC and NL20 cells.

To determine the mechanism of tumor cell growth inhibition by ATV, we performed apoptosis assays on ATV-treated cells. Our results indicated that ATV exposure at 1 μM for 48 h increased the percentages of early and late apoptotic cells in PC-9P (14.7% ± 1.4% versus 2.7% ± 0.3%; p=0.07), PC-9GR (22.7% ± 2% versus 1.1% ± 0.4%; p = 0.004), H1975 (25.9% ± 3.2% versus 3.8% ± 1.1%; p = 0.012) and H1703 (14.1% ± 2.5% versus 2.5% ± 1.3%; p = 0.028), when compared to vehicle (Figure 2D). In NL20 cells, the percentages of apoptotic cells did not differ between vehicle and ATV treatments (3.9% ± 0.7% versus 1.1% ± 0.4%; p = 0.15). These findings show that ATV selectively eradicates cancer cells but has transcurable effects on immortalized non-transformed lung cells.

Statins have been previously shown to inhibit Cav1 expression 29. Flotillin 1 (FLOT1), a lipid raft marker, is utilized as indicator for cholesterol inhibition in western blotting. As shown in Figure 2E, Cav1 and FLOT1 expressions were completely abrogated at 1 μM of ATV in NSCLC and almost completely abolished in NL20 cells, as compared to vehicle. The loss of Cav1 and FLOT1 in ATV-treated cells, at growth inhibitory concentrations, strongly indicate that cholesterol and Cav1 are important for NSCLC cell growth. In NL20 cells, the almost complete loss of Cav1 and FLOT1 proteins and lesser growth inhibition suggest that cholesterol and Cav1 are less crucial for growth of non-transformed lung cells. To exclude the possibility that the inhibition of NSCLC cell growth is not a consequence of ATV off-target effects, we employed another anti-cholesterol drug, Simvastatin, which showed comparable dose-dependent downward trend on tumor cell viability (Figure S3A). Further, western blot data showed that growth inhibition by Simvastatin corresponded with Cav1 downregulation, an observation similar to ATV treatment (Figure S3B).

### Mevalonate supplementation improves growth of ATV-treated NSCLC cells, concomitant with Cav1 re-expression

**Figure 3:**
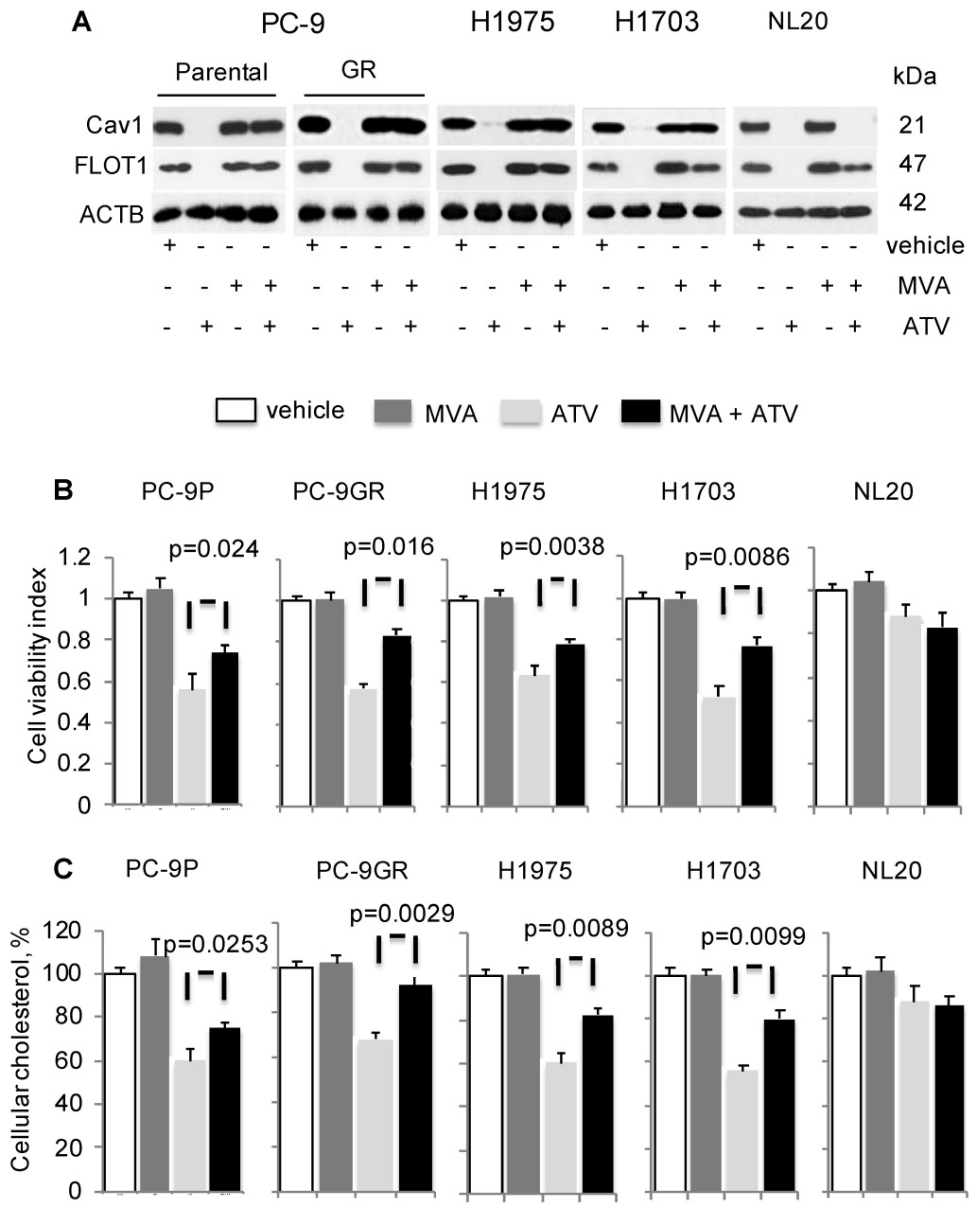
Mevalonate (MVA) supplementation improves growth of Atorvastatin (ATV)-treated NSCLC cells with Cav1 re-expression. Cells were treated with vehicle or 1 μM of ATV for 72 h, or 50 μM of MVA for 48 h, or 1 μM of ATV for 24 h followed by addition of 50 μM of MVA with a further incubation for 48 h. **A.** Western blot analyses demonstrate Cav1 expression. **B.** Cell viability assays (n=3) demonstrate the effect of MVA addition on growth of ATV-treated cells. Significance in differences in cellular cholesterol, in which ATV-treated cells acted as control, was determined by t test. **C.** Total cholesterol measurement in ATV-treated tumor cells with or without MVA supplementation (n=3). Cellular cholesterol is presented as percentages and normalized to vehicle. Significance in differences in cellular cholesterol, in which ATV-treated cells acted as control, was determined by t test.

To determine if cholesterol is important for Cav1 expression and growth of NSCLC, cells were treated with vehicle, ATV, or mevalonic acid (MVA) as single agents, or combination of ATV and MVA. MVA is an intermediate metabolite in cholesterol biosynthesis. Western blotting data demonstrated that MVA supplementation led to Cav1 and FLOT1 protein re-expression in ATV- treated cells, compared to ATV-treated cells without MVA (Figure 3A). In ATV-treated non-transformed immortalized NL20 lung cells, MVA supplementation failed to induce Cav1 re-expression despite FLOT1 re-expression (Figure 3A).

Concomitantly, there was a significant improvement in the viability of ATV-treated NSCLC cells in presence of MVA, as compared to its absence whereas no change in viability of ATV-treated NL20 cells was observed (Figure 3B). PC-9GR cells, however, showed greater viability improvement, compared to PC-9P cells. Specifically, the difference in the improvement of cell viability between PC-9GR and PC-9P cells was significant, as determined by one-way ANOVA (F_1,4_=18.00356, p = 0.01323). Results also showed that the exposure of NSCLC cells to MVA alone did not affect cell viability, compared to vehicle. Total cholesterol measurement revealed that Cav1 re-expression was linked to significant improved levels of cellular cholesterol in ATV-treated cells with MVA as compared to no MVA supplementation (Figure 3C). Importantly, PC-9GR cells showed higher improvement in cellular cholesterol, compared to PC-9P cells, in MVA rescue assays. By comparing the mean values using one-way ANOVA (F_1,4_=38.4391, p = 0.00344), a significant difference was seen in the improvement of cellular cholesterol between PC-9P and PC-9GR cells. No effect was seen on cholesterol levels after the addition of MVA in ATV-treated NL20 cells, when compared to ATV-treated alone (Figure 3C). These data demonstrate there is an oncogenic link between Cav1 and cholesterol in supporting growth of NSCLC but not in non-transformed NL20 cells.

### Cav1 is important for GLUT3-mediated glucose uptake in TKI-resistant NSCLC cells

**Figure 4:**
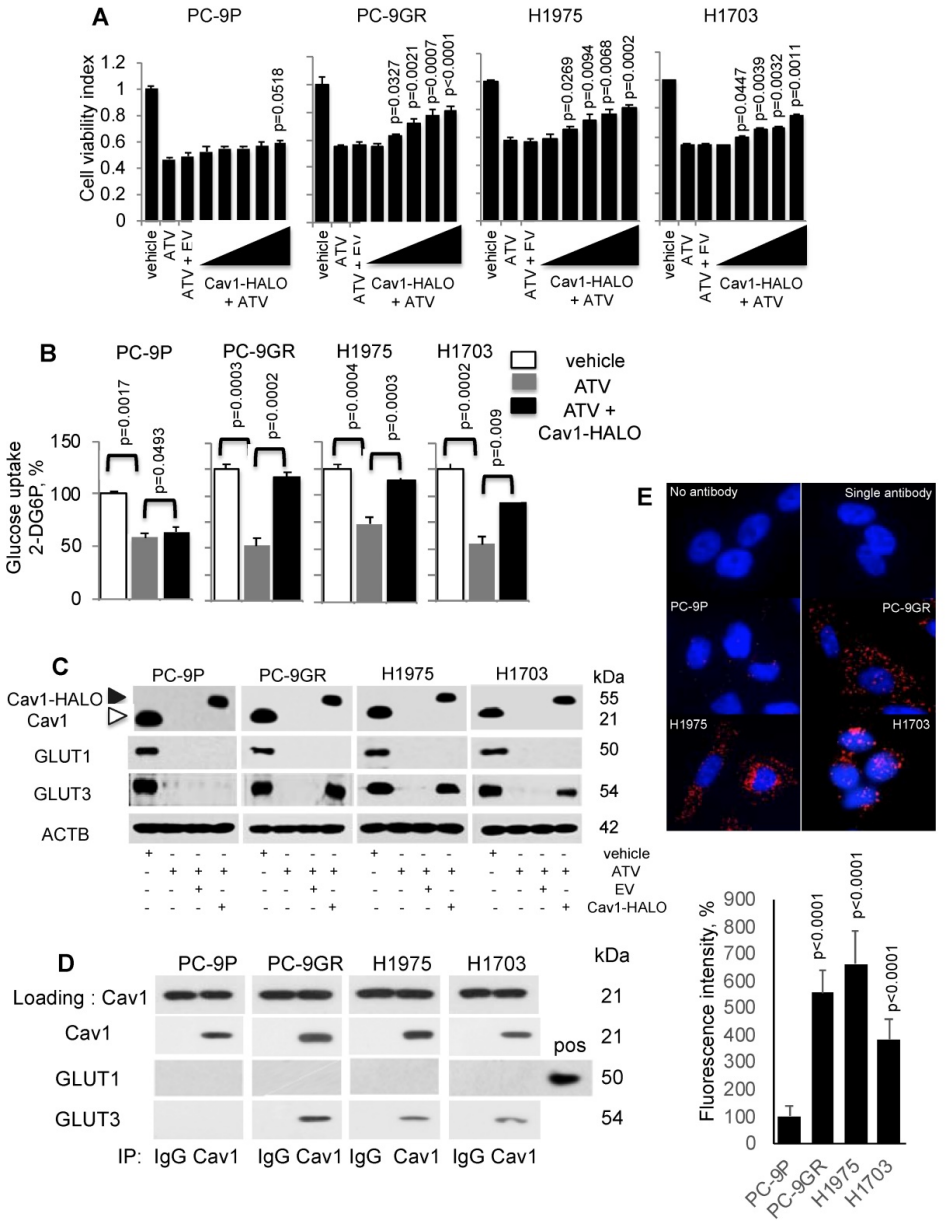
Cav1 improves cellular viability of Atorvastatin (ATV)-treated TKI-resistant NSCLC cells and is associated with GLUT3-mediated glucose uptake. **A.** Cell viability assay (n=3) demonstrates the effects of HALO-tagged Cav1 expression in ATV-treated cells. Cells were seeded in 96-well format prior adding 1 μM of ATV for 12 h. This was followed by transfection of Cav1 constructs ranging from 0.1 to 0.5 μg in increments of 0.1 μg for additional 60 h (total 72 h). Significance in differences in viability indexes, in which vehicle acted as control, was determined by t test. **B.** Glucose uptake measurement (n=3) in tumor cells treated with vehicle, ATV (at 1 μM) or a combination of ATV and Cav1-HALO (2 μg) vector for 72 h using a 6-well plate format. Glucose uptake is presented as percentages and normalized to vehicle. Significance in differences in glucose uptake, in which vehicle or ATV acted as controls, was determined by t test. **C.** Western blotting on Cav1, GLUT1, and GLUT3 protein expression from cells exposed to vehicle, ATV (at 1 μM), ATV and EV (6 ug) combination, or ATV and Cav1-HALO (6 μg) vector transfection combination for 72 h. Black arrow indicates Cav1-HALO, and white arrow indicates endogenous Cav1 proteins. EV - empty vector. Protein size is indicated in kDa. **D.** Cav1 was immunoprecipitated from tumor cell extracts and levels of co-precipitated GLUT1 and GLUT3 was determined with respective antibodies. Protein size is indicated in kDa. **E.** Representative fluorescence images of Duolink Proximity Ligation assays to detect protein interaction between Cav1 and GLUT3 in NSCLC cells. Fluorescence intensities were quantified in 10 cells using Image J software and significance in differences in fluorescence intensities, in which PC-9P acted as control, was determined by t test.

To investigate if Cav1 is important for NSCLC survival, PC-9P, PC-9GR, H1975 and H1703 cells were transfected with HALO-tagged Cav1 (Cav1-HALO) vectors followed by ATV treatment at the clinically relevant dose of 1 μM. Cell viability assays demonstrated that Cav1-HALO expression significantly improved the viability of cholesterol depleted tumor cells when compared to Cav1-depleted ATV-treated cells alone or empty vector-transfected ATV-treated cells (Figure 4A). The viability improvement was weaker in PC-9P cells, compared to PC-9GR cells. Essentially, the difference in improvement of cell viability between PC-9GR and PC-9P cells was significant, as determined by one-way ANOVA (F_1,4_=40.46156, p = 0.00313). The difference in viability improvement between PC-9GR and PC-9P cells suggested that Cav1 had distinct functions in these cells and that Cav1 was more crucial in PC-9GR cell survival. Overall, these results demonstrate that Cav1 is important for the growth and survival of TKI-resistant NSCLC.

Statins have been reported to interfere with glucose metabolism by inhibiting glucose uptake in tumor cells 30, 31. Therefore, we hypothesized that the improved viability of ATV-treated NSCLC cells after Cav1 overexpression is a consequence of unrestrained glucose uptake. To test this hypothesis, cells were incubated with ATV for 72 h followed by measurement of 2-Deoxyglucose (2-DG) uptake using an enzymatic NADPH amplifying system assay. The results demonstrated that glucose uptake was significantly suppressed in ATV-treated cells compared to vehicle (Figure 4B). Cav1 overexpression instead was sufficient to improve glucose uptake in ATV exposed NSCLC cells. Glucose uptake improvement was significantly greater in PC-9GR than PC-9P cells, as determined by one-way ANOVA (F_1,4_=204.84134, p = 0.00014). These data serve as direct evidence that Cav1 is a strong modulator of glucose uptake in TKI-resistant NSCLC while this modulation is much weaker in TKI-sensitive PC-9 cells.

Next, we investigated the effects of Cav1-HALO over-expression on glucose transporter (GLUTs) protein expression in ATV-treated NSCLC cells. Among the 14 GLUTs proteins identified, GLUT1 and GLUT3 have biological significance in NSCLC, in which their overexpression is associated with poor prognosis 32 - 34. Western blot analyses showed that both GLUT1 and GLUT3 were abrogated in ATV-treated NSCLC cells, compared to vehicle (Figure 4C). In addition, our data demonstrate that Cav1 over-expression specifically induces GLUT3 protein re-expression in ATV-treated TKI-resistant but not in TKI-sensitive cells.

To determine whether an interaction between Cav1 and GLUT3 exists in TKI-resistant cells, immunoprecipation assays was carried out using anti-Cav1 antibodies. Western blotting data of pulled-down assays showed that Cav1 interacts with GLUT3, but not GLUT1, in TKI-resistant whereas this interaction was absent in TKI-sensitive PC-9P cells (Figure 4D). These results were verified in reciprocal GLUT3 immunoprecipitation assays as shown in Figure S4. Further, *in situ* Proximity Ligation Assays confirmed physical interactions between Cav1 and GLUT3 in NSCLC cells (Figure 4E). Quantification of fluorescence signals revealed significantly higher readouts in TKI-resistant cells when compared to TKI-sensitive PC-9P cells (Figure 4E). Together, the data demonstrate that an oncogenic physical interaction between Cav1 and GLUT3, and glucose uptake found distinctly in TKI-resistant NSCLC.

### Cav1-GLUT3-mediated glucose uptake is important to maintain cellular energy for TKI-resistant NSCLC survival

**Figure 5:**
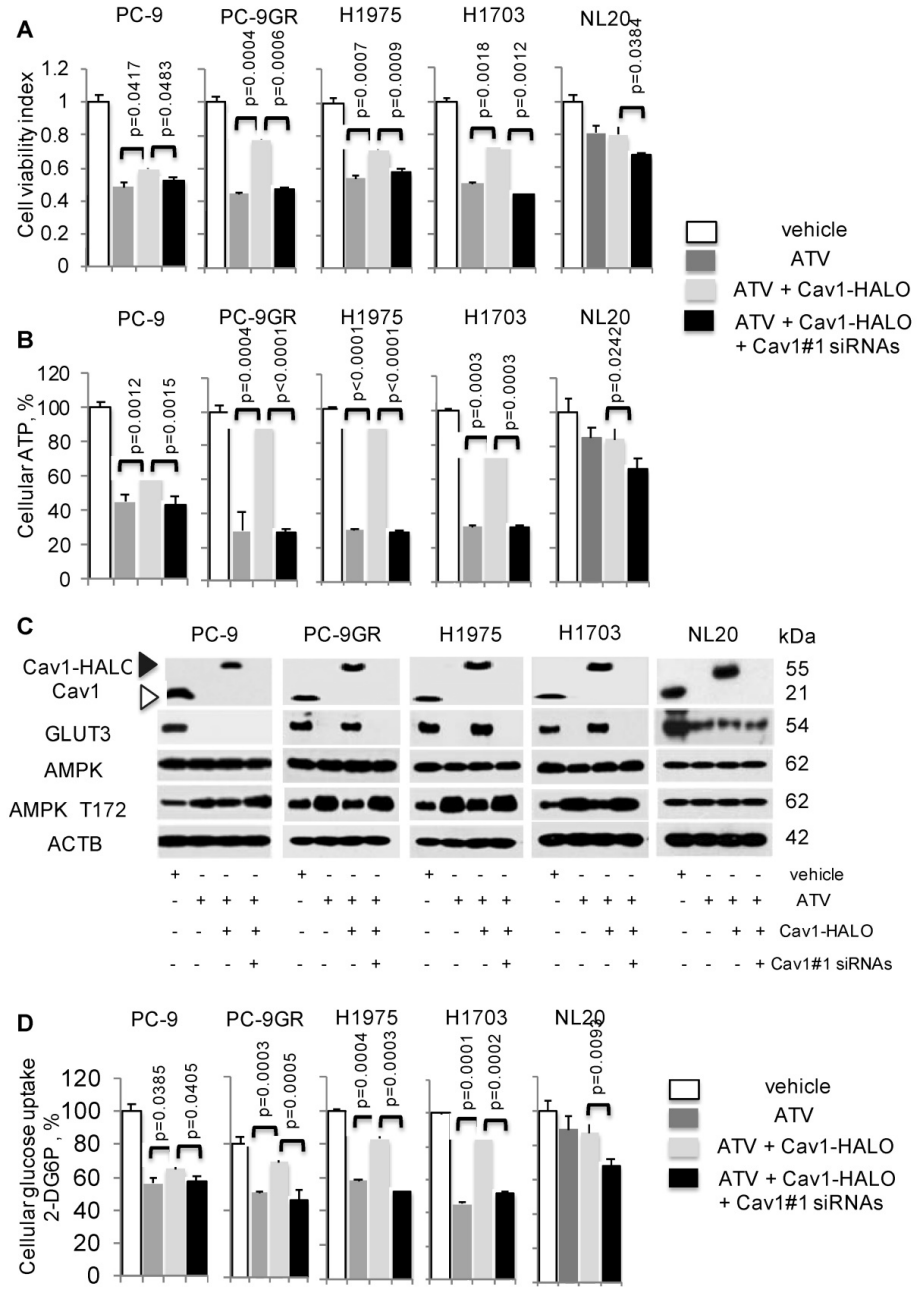
Cav1-GLUT3 signaling is crucial for TKI-resistant NSCLC growth and is associated with cellular ATP production and glucose uptake. **A.** Cell viability (n=3) and **B.** ATP measurement (n=3) assays demonstrate the effects of Atorvastatin (ATV) at 1 μM, combination of ATV and Cav1-HALO (0.5 μg) vector, and combination of ATV, Cav1-HALO, and Cav1#1 siRNAs (25 nM) on tumor cells after 72 h using 96-well format, with vehicle acting as control. Cellular ATP is presented as percentages and normalized to vehicle. Significance in differences in viability indexes and cellular ATP percentages, in which ATV-treated or Cav1-HALO over expressing ATV-treated cells acted as controls, was determined by t test. **C.** Western blotting on Cav1, GLUT3, AMPK-α, and phospho-AMPKα T172, and ACTB protein expression from cells exposed to vehicle, ATV at 1 μM, combination of ATV and Cav1-HALO (6 μg) transfection and combination of ATV, Cav1-HALO and Cav1#1 siRNAs (25 nM) for 72 h. Protein size is indicated in kDa. **D.** Glucose uptake measurement in tumor cells treated with vehicle, ATV at 1 μM or combination of ATV and Cav1-HALO (2 μg) vector for 72 h (n=3). Glucose uptake is presented as percentages and normalized to vehicle. Significance in differences in glucose uptake, in which ATV-treated or Cav1-HALO over expressing ATV-treated cells acted as controls, was determined by t test.

To establish if Cav1 is crucial for NSCLC survival, we examined the consequence of Cav1 knockdown in Cav1-HALO transfected ATV-treated cells by cell viability assays. The efficacies of Cav1 knockdown in these cells were first determined by western blotting (Figure S5). Results from these assays showed that in the context of ATV treatment, TKI-sensitive and -resistant cells displayed a differential effect, in that Cav1 re-expression significantly improved the viability of ATV-treated TKI-resistant cells but showed lesser effect on TKI-sensitive cells (Figure 5A). Specifically, a significant difference was seen in the improved cell viability between PC-9GR and PC-9P cells, as determined by one-way ANOVA (F_1,4_=1161.72, p < 0.00001). In ATV-treated NL20 cells, cell viability, however was unaffected after Cav1-HALO transfection (Figure 5A). Knockdown of Cav1 in CAV1-HALO transfected ATV-treated NL20 cells showed a weaker reduction in cell viability, an observation similar to that in TKI-sensitive PC-9P cells, when compared to TKI-resistant cells. These results indicate that Cav1 is not crucial for non-transformed lung cell growth. Together, the data demonstrate that TKI-resistant NSCLC cells exhibit a greater reliance on Cav1 for survival than TKI-sensitive NSCLC and non-transformed lung cells.

Cellular ATP was measured to establish the link between Cav1 and cellular energy. ATV exposure led to reduced cellular ATP in NSCLC cells (Figure 5B). In TKI-resistant cells, Cav1 over-expression in ATV-treated cells led to a significant improvement of cellular ATP levels whereas in TKI-sensitive PC-9P, the increase of ATP levels was weaker, as determined by one-way ANOVA (F_1,4_=617.79, p = 0.000016). Inhibition of Cav1-HALO overexpression by siRNAs in ATV-treated cells, abrogated the improved ATP levels to levels comparable to ATV-treated cells alone. Cellular ATP levels, however remained unchanged after CAV1 overexpression, and the subsequent Cav1 knockdown led to a weak ATP inhibition in ATV-treated NL20 cells, (Figure 5B). These results show that Cav1 plays an important role in regulating cellular energy in TKI-resistant rather than TKI-sensitive NSCLC and non-transformed lung cells.

The AMP-activated protein kinase (AMPK) is an important regulator of cellular function in response to cellular energy stress 35, 36. Under conditions that induce energy fluctuations in cells, AMPK undergoes a conformational change and exposes its phosphorylation active site, threonine 172 (T172) located on the α subunit. Western blotting demonstrated that ATV treatment led to an elevation of AMPK-α T172 phosphorylation accompanied by complete loss of Cav1 and GLUT3 expression in TKI-sensitive and -resistant NSCLC cells, compared to vehicle treated cells (Figure 5C). Transfection of with Cav1-HALO vectors resulted in reduced AMPK-α activity concomitant with the up-regulation of GLUT3 expression in ATV-treated TKI-resistant but not TKI-sensitive cells, compared to cells with ATV treatment alone (Figure 5C). However, phosphorylated AMPK-α T172 levels did not differ much between ATV-treated and Cav1-HALO transfected ATV-treated TKI-sensitive PC-9P cells. Forced Cav1-HALO knockdown abrogated Cav1-HALO and GLUT3 expression while phosphorylated AMPK-α T172 was elevated in ATV-treated TKI-resistant cells when compared to Cav1 knockdown ATV-treated cells (Figure 5C).

In NL20 cells, AMPK-α activity and Cav1 levels were unaffected, while GLUT3 was decreased after ATV exposure compared to vehicle (Figure 5C). AMPK-α activity and GLUT3 levels remained unchanged after Cav1 overexpression in ATV-treated NL20 cells. Further, Cav1 knockdown in Cav1 expressing ATV-treated NL20 cells affected neither GLUT3 nor AMPK activity. Together, these data demonstrate that Cav1 correlates with GLUT3 expression in TKI-resistant NSCLC cells. Fluctuations in phosphorylated AMPK-α T172 levels indicate that Cav1 signaling plays an important role in maintaining cellular energy in TKI-resistant cells but not in TKI-sensitive NSCLC and non-transformed lung cells.

Glucose uptake assays were then carried out, in which the uptake and accumulation of 2-deoxyglucose-6-phosphate (2-DG6P) in cells was measured via detection of nicotinamide adenine dinucleotide phosphate (NADPH) produced from the oxidation of 2-DG6P in the presence of glucose-6-phosphate dehydrogenase (G6PDH). Results from glucose uptake assays demonstrated that glucose uptake was significantly reduced in both ATV-treated TKI-sensitive and -resistant cells, compared to vehicle (Figure 5D). Cav1-HALO expression induced a significant improvement of glucose uptake in TKI-resistant but had a lesser effect in TKI-sensitive ATV-treated cells, as determined by comparing the mean values using one-way ANOVA (F_1,4_=422.5, p = 0.000033). The inhibition of Cav1-HALO expression by Cav1#1 siRNAs reversed the improved glucose uptake in TKI-resistant ATV-treated cells. These results demonstrate that glucose uptake is tightly coupled with Cav1 in TKI-resistant cells whereas the coupling is weaker in TKI-sensitive NSCLC cells. Conversely, there was no significant change of glucose uptake in NL20 after ATV exposure and Cav1-HALO transfection, while Cav1 silencing in Cav1 expressing ATV-treated NL20 cells led to a weaker drop of glucose uptake (Figure 5D). Further, cell viability assays showed that restricting concentrations of exogenous glucose worsened the improved viability seen in Cav1-HALO expressing ATV-treated cells thus suggesting the importance of glucose for the survival of NSCLC cells (Figure S6). Collectively, our data suggest Cav1 signalling is important in cellular energy regulation in TKI-resistant but less so in sensitive NSCLC and non-transformed lung cells. More importantly, we demonstrate that modulation of glucose uptake via GLUT3 by Cav1 is more apparent in TKI-resistant than -sensitive NSCLC and non-transformed lung cells, which suggest distinct Cav1 function and different metabolic requirements between these cells.

### Atorvastatin delays growth of NSCLC cell line-derived xenografts and EGFR-T790M-L858R transgenic models

**Figure 6:**
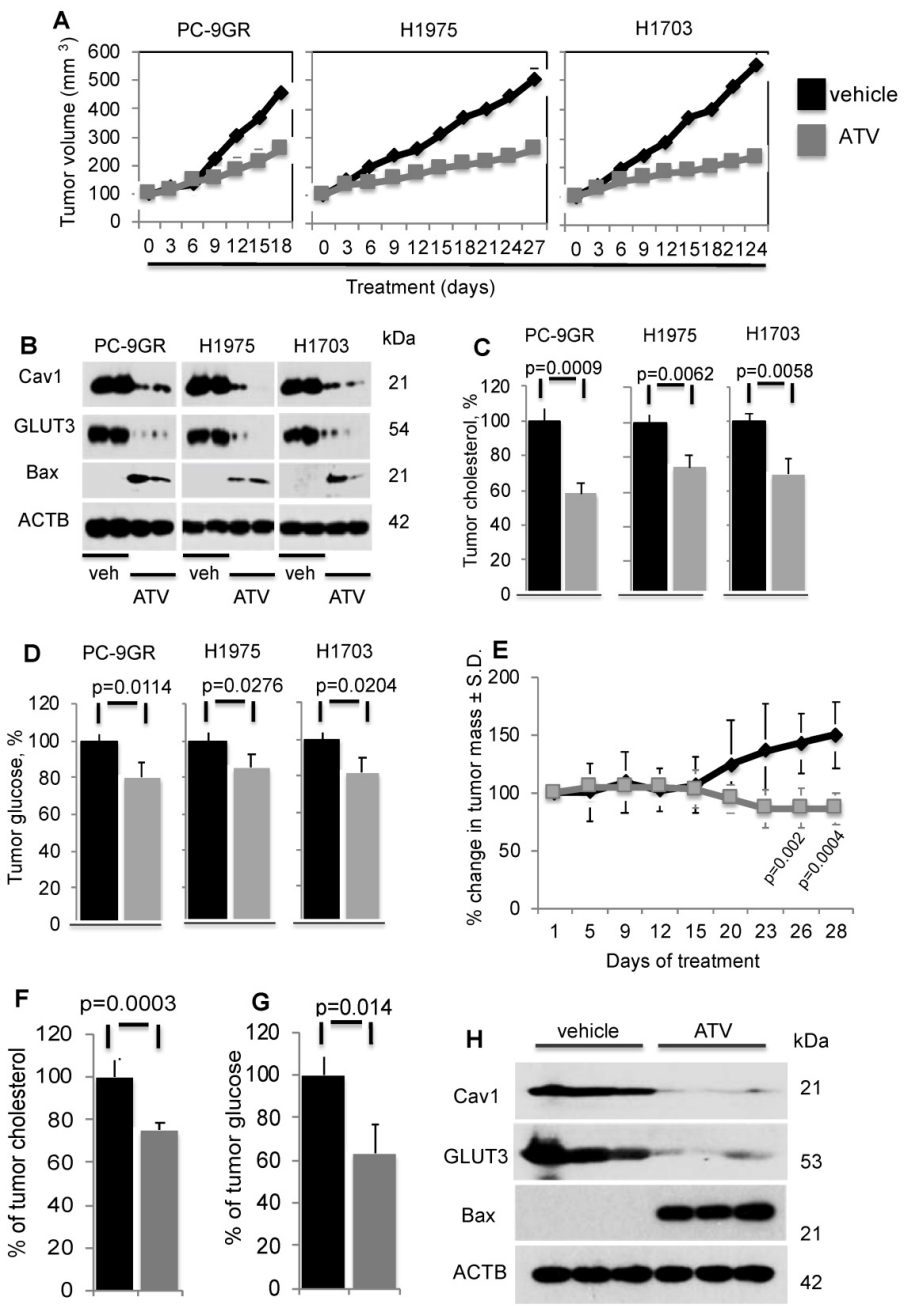
Atorvastatin (ATV) demonstrates anti-tumor activity in NSG xenografts and EGFR T790M/L858R transgenic mouse models. **NSG model.** Tumor reduction correlates with loss of Cav1 and GLUT3, up regulation of Bax proteins, and significantly lower tumor cholesterol and glucose content. **A.** Tumor growth curve of TKI-resistant NSCLC cell lines xenografted into NSG mice, subsequently treated with either vehicle or ATV at 30 mg/kg body weight (upper panels). Representative images of xenografts from vehicle- and ATV-treated mice (lower panels). **B.** Western blotting demonstrate Cav1, GLUT3 and Bax expression in protein extracts from two xenograft tumors from each cell line, treated with vehicle- or ATV. Protein size is indicated in kDa. **C.** Tumor cholesterol and **D.** Glucose content measurements in tumors from vehicle- and ATV-treated mice (n=5 each group). Tumor cholesterol and glucose content are presented as percentages and normalized to vehicle. Significance in differences in tumor cholesterol and glucose content, in which vehicle acted as control, was determined by t test. **Transgenic model E.** Graph demonstrates percentage of change in tumor volume between ATV- (n=7) and vehicle-treated (n=6) groups. Significance in differences in tumor volumes, in which vehicle acted as control, was determined by t test. **F.** Measurement of cholesterol from tumors of vehicle- or ATV-treated transgenic mice (n=5 for vehicle and n=5 for ATV groups). Significance in differences in tumor cholesterol levels, in which vehicle acted as control, was determined by t test. **G.** Measurement of glucose from tumors of vehicle- or ATV-treated transgenic mice (n=5 for vehicle and n=5 for ATV groups). Significance in differences in tumor glucose content, in which vehicle acted as control, was determined by t test. **H.** Western blot showing Cav1, GLUT3 and Bax expressions in protein extracts from representative tumors from vehicle- and ATV-treated groups.

To determine ATV's efficacy as anti-tumor agent *in vivo*, xenograft models of PC-9GR, H1975 and H1703 cells were generated in NSG mice. In Figure 6A, tumor growths in daily ATV-treated (at 30mg/kg) 37, 38 mice (n=5 mice) were significantly slower compared to their vehicle counterparts (n=5 mice), with p-value of 0.0057, 0.0046, and 0.002 for PC-9GR, H1975 and H1703 xenografts, respectively. Mice were euthanized at day 18, 27 and 24 for PC-9GR, H1975, and H1703 xenografts respectively, as tumors in vehicle group reached the maximum size allowed (NUS IACUC guidelines). Tumors in treatment groups were smaller than those in vehicle groups as shown in Figure 6A. Throughout the course of ATV administration, we did not observe any obvious side effects or changes in body weight (Figure S7). Western blotting analyses on tumor protein extracts from ATV and vehicle groups indicated that ATV inhibited Cav1 and GLUT3 expression while pro-apoptotic Bax was up regulated, compared to vehicle (Figure 6B). Tumor cholesterol was then measured and compared between ATV and vehicle groups (5 mice per group). Results demonstrated significant reduction of tumor cholesterol in tumors from the ATV group compared to vehicle (Figure 6C). Further, glucose measurement revealed significant glucose reduction in tumors compared to vehicle (Figure 6D). Taken together, the data show that ATV exhibits anti-tumor activity, and lowers tumor cholesterol and glucose in TKI-resistant NSCLC *in vivo*.

The *in vivo* efficacy of ATV was further examined in the transgenic CCSP-rTTA-EGFR L858R-T790M-driven lung adenocarcinoma mouse model 39, 40. In these mice, lung tumors develop when continuously exposed to doxycycline, as a result of mutant EGFR overexpression in CCSP^+^ pulmonary epithelial cells. Lung tumor growth was detected and closely monitored by magnetic resonance imaging (MRI), and baseline MRI demonstrated tumor growth in the lungs after 5-6 weeks of induction. At such time point, mice were randomized to vehicle (n=6) or ATV (n=7) treatment. MRI images were then taken every 2 to 4 days to capture the effects of drug treatment on tumor size over a 28-day period. Processing and quantification techniques of tumor burden were based on manual segmentation/volume calculation of diffuse lung tumors as described previously 41. Changes in tumor volumes over the course of treatment were calculated as percentage change in volume over tumor volume at day 1 of treatment, which was set at 100% (Figure 6E).

There was no significant difference in tumor volumes between vehicle and ATV groups from day 1 to day 15 of treatment. All mice were euthanized at treatment day 28 when tumor burden of vehicle-treated mice necessitated euthanasia given their poor body conditions. Importantly, at treatment days 26 and 28, lung tumors in the ATV-treated group were found to be significantly smaller by approximately 33% than those in the vehicle-treated set (Figure 6E). Tumor cholesterol percentages demonstrated that the tumor shrinkage in the ATV-treated group correlated with significantly lower tumor cholesterol when compared to vehicle (Figure 6F). Measurement of tumor glucose demonstrated that the reduced tumor sizes in ATV-treated group correlated with significantly lower glucose content when compared to vehicle (Figure 6G). Subsequently, western blot images showed Cav1, GLUT3 and Bax expressions in tumors from ATV and vehicle-treated transgenic mice (Figure 6H). Our data demonstrated that tumor growth inhibition correlated with reduced Cav1 and GLUT3 expressions, while Bax was up regulated when compared to vehicle (Figure 6H). Altogether, our data demonstrate the *in vivo* anti-tumor activity of Atorvastatin as a single agent against the clinically relevant TKI-resistant EGFR T790M/L858R mutation.

## Discussion

Although the role of cholesterol in cancer remains elusive, there are increasing lines of evidence, shown in both cell culture systems and *in vivo*, which demonstrate the benefits of statin therapy in the treatment of various carcinomas including prostate, melanoma, renal, breast, and colorectal 42- 49. The mechanisms by which statins exert their anti-neoplastic properties are diverse 16 - 18, 50. In the current study, we identified a novel link between TKI-resistance and elevated cellular cholesterol in NSCLC. This association is uncovered in cells carrying wild-type and mutated EGFR. A plausible explanation is that this observation is a consequence of metabolic reprogramming in malignant cells, and is an EGFR-independent mechanism. Further, we demonstrate that combination therapy of ATV and Gefitinib reverses induced EGFR-TKI resistant EGFR mutated (PC-9GR and H1975) but not in native EGFR-TKI resistant EGFR wild-type NSCLC cells (H1703). This pre-clinical data shows the prospect of ATV in circumventing acquired EGFR-TKI resistance in NSCLC. Apart from cholesterol inhibition, statins inhibit cholesterol-independent processes including cellular proliferation and disrupt intracellular signaling 17, 51.

Our data demonstrate that ATV lowers cellular cholesterol, induces loss of Cav1 expression and suppresses growth of NSCLC cells by triggering apoptosis. Mevalonate supplementation demonstrates that cholesterol is important for Cav1 expression in tumor cell survival. Reconstitution of Cav1 in Cav1-depleted NSCLC cells reveals a strong oncogenic role of Cav1 4, 5, 52 in TKI-resistant NSCLC but not in non-transformed pulmonary NL20 cells. Though ATV could suppress growth of TKI-sensitive cells, the weaker viability improvement in TKI-sensitive cells demonstrates that Cav1's role is less important and the growth inhibition by ATV in TKI-sensitive cells occurs through suppression of unknown oncogenic proteins that these cells rely on growth and survival. Recent studies investigating the roles of cellular Cav1 in liver and endothelial cells have reported similar findings, thus suggesting that Cav1 is functionally relevant in the regulation of cellular growth 12, 13.

Importantly, our findings have led us to believe that we have identified a link between cholesterol and Cav1, which is important for the survival of TKI-resistant NSCLC cells, and that can be targeted by statins. We demonstrate that Cav1 re-expression augmented glucose uptake in Cav1-depleted TKI-resistant tumor cells, an observation consistent with an earlier study on *Cav-/-* mice whereby glucose supplementation led to an improved liver regeneration after partial hepatectomy 12. Our data demonstrate that there is a greater dependency on Cav1 for survival in TKI-resistant rather than TKI-sensitive NSCLC and non-transformed lung cells, suggesting that Cav1 has distinct functions in these cells.

Glucose transport is facilitated by glucose transporters (GLUTs) to carry extracellular glucose across cell membranes and is crucial for cellular growth and proliferation. Our findings demonstrate that Cav1 mediates glucose uptake via GLUT3 in TKI-resistant but not in TKI-sensitive tumor cells. Importantly, our immunoprecipitation data demonstrate that the interaction between Cav1 and GLUT 3 found in TKI-resistant cells only. These differences suggest that TKI-sensitive and -resistant tumor cells possess different metabolic requirements for growth and survival, and that the Cav1-GLUT3 axis is distinct in TKI-resistant cells. GLUT3 has a higher affinity for glucose than GLUT1, GLUT2 and GLUT4, and exhibits greater transport capacity than GLUT1 and GLUT 4 53. Further, GLUT3 is reported to possess the highest turnover rate among all GLUT family members 54, implying that glucose transport activity is important to meet the greater energy requirements for tumor cell growth. Hence, its expression provides strong evidence on the high glucose demand to support NSCLC growth 33, 55. The loss of GLUT3 expression, as a consequence of Cav1 depletion, results in growth inhibition and cellular energy reduction. These results demonstrate that Cav1-GLUT3 mediated glucose uptake is crucial for cell energy homeostasis in TKI-resistant tumor cells.

A possible explanation for the discrepancy observed in the response between EGFR mutated TKI-sensitive PC-9P and TKI-resistant PC-9GR to Cav1 inhibition, may be acquisition of additional oncogenic changes in PC-9GR cells that may converge to tighten the Cav1-GLUT3 axis, which is also present in TKI-resistant EGFR mutated (H1975) and wild-type (H1703) NSCLC cells. Tightening of the Cav1-GLUT3 axis occurs possibly due to changes in the physical properties of Cav1 favoring GLUT3 binding as demonstrated in Proximity Ligation Assays where significantly stronger Cav1 and GLUT3 physical interactions were observed in TKI-resistant than -sensitive NSCLC cells. The absence of Cav1-GLUT3 signaling axis in TKI-sensitive tumor cells indicates that this oncogenic pathway is distinct in TKI-resistant NSCLC. Apart from the identification of Cav1-GLUT3 interactions, there are currently no markers to predict response to statins in TKI-resistant NSCLC. In recent years, there has been ongoing growing interest to identify genetic variations/polymorphisms towards the variations in response to statins through pharmacogenetics studies 56-58. The identification of new candidate genes, through these studies, can aid in the selection of patients with genotypes that will benefit from statin treatment.

The efficacy of ATV as anti-tumor agent was subsequently tested through *in vivo* xenograft assays in NSG mice. Daily ATV administration suppressed tumor growth in mice carrying TKI-resistant PC-9GR, H1975, and H1703 xenografts, by 59%, 48%, and 57%, respectively, compared to their vehicle counterparts. The reduced tumor sizes from ATV treatment corresponded to loss of Cav1 and GLUT3, induced pro-apoptotic Bax, and lowered tumor cholesterol content. Further, glucose levels were significantly reduced following ATV treatment, compared to vehicle treatment, which supports our cell culture data. The anti-tumor activity of Atorvastatin in vivo was further verified in a transgenic mouse model expressing the clinically relevant EGFR T790M/L858R mutation, in which at treatment termination, Atorvastatin-exposed animals presented with approximately 33% decrease in tumor mass (assessed by MRI) as compared to vehicle-treated transgenic mice.

**Figure 7:**
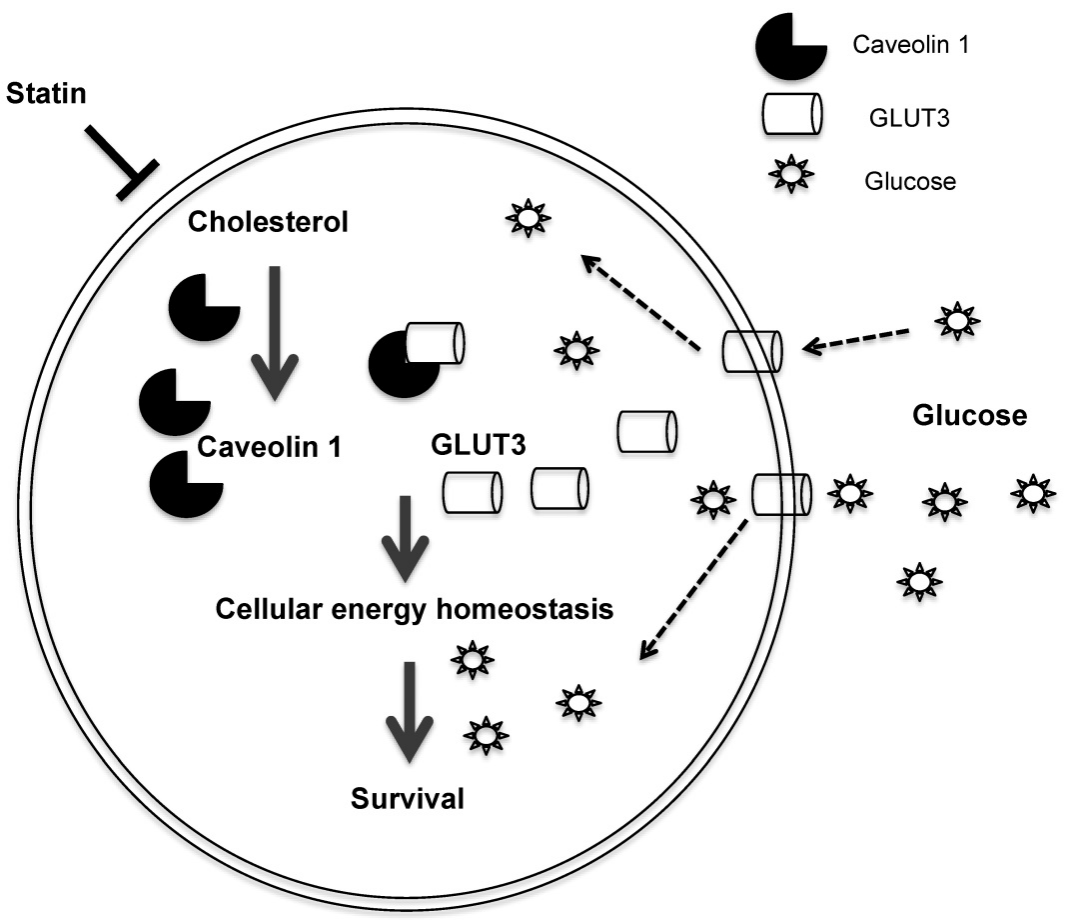
Schematic diagram of the proposed model depicting how Atorvastatin, an anti-cholesterol drug, suppresses growth of TKI-resistant NSCLC tumors. Atorvastatin inhibits Cav1, which regulates GLUT3 signalling, supports tumor growth by modulating cellular energy homeostasis through glucose uptake.

In summary, we uncovered a mechanism by which the statin ATV inhibits Cav1 expression, thereby suppressing growth of TKI-resistant NSCLC (Figure 7). Cav1 reconstitution in Cav1-depleted NSCLC cells demonstrates that Cav1 exerts its oncogenic effect through the regulation of glucose uptake via GLUT3, which is important to maintain the energy demand for tumor growth. Additionally, the differential effects of atorvastatin shown between EGFR-TKI resistant and sensitive cells indicate that EGFR mutation status may influence its actions. Our data support the promise that statins are candidate drugs for TKI-resistant NSCLC treatment. More importantly, our pre-clinical *in vivo* data offer compelling evidence to repurpose statins, which is a commonly available drug, from the conventional anti-cholesterol agent to its application in cancer therapy. One major limitation of oral statin delivery however is the low systemic bioavailability and this may have contributed to inconsistent reports in clinical studies examining its protective role in lung cancer patients 20-23, 59-61. Hence, alternative routes of statin administration are warranted to improve its efficacy. The repurposing of statins, as an inhaled therapy, is one such strategy as it delivers higher concentrations of drug to the lungs at lower drug dosages as compared to oral delivery 62, 63. There are increasing lines of evidence showing the potential and benefits of using inhaled statins in mouse models with inflammatory lung diseases 64-66. Further investigations are needed to translate the inhaled formulation to the clinical setting for the treatment of NSCLC with EGFR-TKI resistance.

## Materials and Methods

### Reagents

The target sequences of short-interfering RNAs for EGFR (5' -CAGGAACTGGA TATTCTGAAA-3') 67, Cav1#1 (5'-CACCTTCACTGTGACGAAATA-'3) and Negative (scrambled) Control (5'-AATTCTCCGAACGTGTCACGT-'3) were purchased from Qiagen. Anti-Cav1 (rabbit sc-894, 1:1000 and mouse sc-53564, 1:1000), GLUT1 (sc-377228, 1:1000), GLUT3 (sc-74497, 1:1000), FLOT1 (sc-74566, 1:1000) and β-actin (ACTB, sc-47778, 1:2000) antibodies were purchased from Santa Cruz Biotechnology (Santa Cruz, CA). Anti-total AMPKα (#5831, 1:1000), phospho-AMPKα T172 (#2535, 1:1000) and Bax (#2772, 1:2000) antibodies were purchased from Cell Signaling. Mevalonate (#4667) was purchased from Sigma. HALO-tag expression vectors pFN21A-CAV1 and pFN21A were purchased from Promega. Atorvastatin (ATV; Lipitor) and Simvastatin (Zocor) were purchased from Pfizer and MSD Pharmaceuticals Singapore respectively. Both drugs were dissolved in DMSO for cell culture experiments. For *in vivo* models, ATV was dissolved in sterile 0.9% NaCl solution.

### Cell culture

Human NSCLC cells, PC-9 (EGFRdelE746-A750), HCC827 (EGFRdelE746-A750), H1975 (EGFRL858R/T790M) and H1703 (EGFR wild-type), and immortalized non-transformed NL20 lung cells were purchased from the American Type Culture Collection. These cells tested negative for mycoplasma (Figure S8; MycoAlert assay from Lonza LT07-218) and were authenticated via DNA fingerprinting (Figure S9). These cells were maintained in either RPMI or MEM (Gibco) supplemented with 10% FBS (Hyclone), 2g/L glucose and penicillin/streptomycin (P/S). All cell lines were grown at 37°C under 5% CO_2_. Cells were grown to ~80% confluence, harvested with trypsin, and suspended to the cell density required for each assay. PC-9 Gefitinib resistance (PC-9GR) was generated through chronic exposure of drug at gradual dose increments (from 50 nM up to 5 μM) for 6 months 67. The IC50 of PC-9GR was determined to be about 3 μM (versus 50 nM for parental PC-9) and cells were then maintained in 1 μM of Gefitinib prior to all experiments. Further, DNA from PC-9GR were analysed to rule out the relation between the development of acquired resistance to Gefitinib of PC-9GR cells and secondary EGFR T790M mutation or MET gene amplification.

### Cell viability and proliferation assays

Cell viability and proliferation were evaluated using the 3-(4,5-dimethylthiazol-2-yl)-5-(3-carboxymethoxyphenyl)-2-(4-sulfophenyl)-2H-tetrazolium) (MTS, G3580, Promega) and 5-bromo-2'-deoxyuridine (BrdU, #6813, Cell Signaling) assays, respectively. Cells were plated in 96-well plates at a density of 3,000 - 4,000 cells/well in complete medium for 24 h. Cells were then treated with respective agents the following day. All samples were assayed in quadruplicates.

### Gene knockdown assays

Cells were grown to 50-60% confluence and transfected with 25 nmol/L of siRNAs using liposomes (Lipofectamine RNAiMax, #13778, Invitrogen) for 72 h. For negative control, cells were transfected with scrambled siRNAs.

### Vector transfection assays

Transfection of HALO-tag CAV1 expression vectors into cells were performed using Fugene HD (E2311, Promega) following the manufacturer's instruction. Briefly, cells (50-60% confluence) were seeded in 6-well plates and allowed to attach overnight followed by incubation with DNA-Fugene HD mixture comprising 1:6 of DNA : Fugene HD reagent ratio diluted in OPTI-MEM (Invitrogen) for 48 to 72 h. Cells transfected with pFN21A vectors acted as controls. Transfection efficiency was determined by protein expression.

### Immunoblot assays

Post-treatment cultured cells in 10 cm dishes were collected at specific times and solubilized in Cellytic buffer (C2978, Sigma) with protease and phosphatase (Roche) inhibitor cocktail. Proteins were separated by SDS-PAGE, transferred to PVDF membranes (Thermo) and detected using Western Bright ECL (Advansta, CA).

### Immunoprecipitation

Harvested cells were lysed in ice-cold IP buffer [150 mM NaCl, 0.5% NP40, 1 mM EDTA, 20 mM Tris (pH 7.4), 1 mM NaF, 1 mM Na2VO4, 20 mM b-glycerolphosphate, protease inhibitor cocktail] by passing through 23G needle ten times. The lysates were then cleared by centrifugation and subsequently incubated with mouse anti-Cav1 or rabbit anti-GLUT3 or respective control IgG antibodies for 2.5 h with rotation at 4°C. Protein G beads were then added followed by 1.5 h rotation at 4°C. After incubation, samples were rinsed three times with IP buffer and immunoprecipitates were resolved on SDS-PAGE gel, and GLUT3 and Cav1 were detected by western blotting.

### Apoptosis assays

Flow cytometric analyses were carried out on single-cell suspensions and were analyzed by FACScan using CellQuest software (BD Biosciences, San Jose, CA). Apoptosis assay was performed with an Annexin V-FITC Apoptosis Detection Kit (BD Biosciences, San Jose, CA). Briefly, harvested cells were suspended in 100 μl of the binding buffer followed by additions of 5 μl Annexin V-FITC and 5 μl propidium iodide (PI, 10 mg/mL) and samples were incubated for 15 min at room temperature in dark. Finally, binding buffer (400 μl) was added to each reaction tube and cells were analyzed using CellQuest software (BD Biosciences, San Jose, CA).

### Duolink In situ Proximity Ligation Assays

Samples were prepared according to manufacturer guidelines (Duolink In Situ PLA Kit, #DUO92101 Sigma-Aldrich). Briefly, cells were grown on glass coverslips overnight and followed by fixation in 4% paraformaldehyde for 15 min at room temperature. Fixed cells were permeabilized, blocked and subsequently probed with anti-Cav1 (1:100 dilution, Mouse, MINUS) and -GLUT3 (1:100 dilution, Rabbit, PLUS) primary antibodies for 2 h at room temperature. Samples were then incubated with a pair of PLA probes (one PLUS and one MINUS) and signal development (ligation, amplification, and hybridization) was performed according to the manufacturer's instructions. Fluorescence signals were observed with a confocal microscope (Bio-Rad/Zeiss) and quantified using the ImageJ software (analyze particles plug-in). Percentages of fluorescence intensities of Cav1 and GLUT3 interactions were determined in 10 cells and plotted for TKI-resistant cells with TKI-sensitive PC-9P cells as control.

### Glucose uptake assays

2-Deoxyglucose uptake was performed by an enzymatic NADPH amplifying system assay (ab136955, Abcam, UK) following the manufacturer's instruction. Briefly, 3,000 cells were seeded in 96-well format overnight and exposed to indicated agents for 72 h. Treated cells were counted and normalized for cell number, washed with 100 μl of Krebs-Ringer-Phosphate-HEPES (KRPH) buffer containing 2% BSA for 40 min, stimulated with 1 μM insulin for 20 min to activate glucose transporters and followed by the addition of 10 mM 2-deoxyglucose to wells for 40 min. Cells were then washed 3 times with PBS, and lysed to prepare a 50 μl reaction system. Glucose uptake in samples were measured at OD412nm and quantified against a standard curve generated using 2-DG6P standard solution.

### Glucose assays

Tumor glucose content was measured following the protocol of Glucose assay kit (ab65333, Abcam). Briefly, 10 mg of tumor was harvested, rinsed 3 times with ice-cold PBS and homogenized in 100 μl Assay buffer on ice. Samples were centrifuged at 12,000 x g for 5 min at 4°C. Supernatant was transferred to a new tube followed by deproteinization with 1M perchloric acid (PCA) and 50 μl reaction system was prepared. Glucose content in samples were measured at OD570nm and quantified against a standard curve generated using 1 mM glucose standard solution.

### Cellular ATP assays

Cells were treated with respective agents in 6-well dish format followed by harvesting and subsequent homogenization in 100 μl of assay buffer. Cells were normalized for cell number and cellular ATP were measured by a colorimetric assay following the protocol of ATP assay kit (ab83355, Abcam). ATP concentrations in the samples were determined at 570 nm in a microplate reader against a standard curve generated using 1 mM ATP standard solution.

### Cholesterol measurement assays

Cholesterol was measured using cholesterol/cholesteryl ester quantitation kit (ab102515; Abcam) following manufacturer's protocol. Briefly, lipids were extracted from 1 x 10^6^ cells or 10 mg tumor with 200 μl CHCl3:IPA:NP-40 (7:11:0.1) using a micro-homogenizer. Sample extracts were then centrifuged for 5 min at 15, 000 x g. The liquid phase was transferred to a new tube and dried at 50°C for 30 min using a speedvac. Samples were then suspended in 200 μl Assay buffer and 50 μl reaction mixtures were prepared. Cholesterol content in samples was determined at 450 nm against a standard curve generated using 0.25 mg/ml cholesterol standard solution.

### Xenograft models

Animal experimental protocols were approved by the NUS Institutional Animal Care and Use Committee (Protocol R14-579). Male NOD scid gamma (NSG) mice were obtained from the Xenograft Cancer Model Facility, Cancer Science Institute Singapore and maintained in the National University of Singapore Animal Facility throughout this study. PC-9GR, H1975 and H1703 NSCLC cells were selected for injection. Mouse numbers were determined using the formula n = log / log p, where n = number of animals that need to be tested = the probability of committing a Type II error / p = the proportion of the animals in the colony without tumor growth 68. For our study, we use = 0.05, as we wish to have a 95% chance of detecting the event of tumor formation and we expect the incidence of tumor formation to be 50% in our study. Hence, p = 0.50. n = log / log p = log 0.05/ log 0.50 = 4.33. Mice were randomized, in which even numbered mice were allocated to vehicle group whereas odd numbered mice were allocated to ATV group (n=5 for each group). Experimenters were blinded to the treatment groups to avoid bias. Cells were suspended at 3x10^6^ in 200μL of 1 part PBS: 1 part Matrigel solution (Corning, MA), and implanted into the right flank of 6- to 8-week-old NSG mice, using a syringe with a 27 gauge disposable needle (BD Biosciences). Mice were previously anesthetized with isoflurane by inhalation at 3-4% (Baxter, IL). When tumors reached approximately 4 mm in diameter, mice were treated with either ATV (30 mg/kg) or vehicle by intraperitoneal injections for 5 consecutive days per week. For mice injection, ATV was prepared in sterile 0.9% NaCl. Tumors were measured with caliper every 3 days and their volumes calculated using the formula volume = 0.5 x length x width^2^. Mice were then sacrificed by CO_2_ inhalation when tumor reached a diameter of 15mm in the vehicle group or presented with ulceration at the tumor site. Tumors were harvested and snap frozen in liquid nitrogen and stored at -80°C.

### CCSP-rtTA-EGFRT790M/L858R Transgenic models

All animal work was carried out in specific pathogen-free housing with abundant food and water under guidelines approved by the BIDMC Institutional Animal Care and Use Committee and Animal Research Facility. CCSP-rtTA tetracycline-dependent activator mice and tet-regulatable EGFRL858R+T790M mice have been previously described, were a kind gift from Dr. Kwok-Kin Wong, and were maintained on a C57BL/6 genetic background. Calculations to determine mouse numbers are similar to the formula used for Xenograft model. However, we expect the incidence of tumor formation to be 40% in the transgenic model. Hence, p = 0.60. n = log / log p = log 0.05/ log 0.60 = 5.86. To induce EGFR expression, doxycycline was administered by feeding both male and female mice with doxycycline-impregnated food pellets (625 ppm; Harlan-Teklad). 8 to 10 weeks old mice were fed with doxycycline to induce lung tumors. Mice were then imaged by magnetic resonance (MRI) to detect baseline tumors, and subsequently recruited into treatment groups. Utilizing randomization process similar to that for Xenograft model, 6 (2 males and 4 females) and 7 (3 males and 4 females) mice were allocated to vehicle and ATV treatment groups respectively. Experimenters were blinded to the treatment groups to avoid bias. MRI images of mouse lungs were captured with a Bruker Biospec 94/20 9.4 Tesla scanner and the primary imaging sequence used was RARE (Rapid Acquisition with Refocused Echoes), with TR/TE=1200ms/17.5ms.

### Statistical analysis

Results from all assays were from at least two independent experiments and each performed in triplicates, and is shown as means ± s.e.m. All statistical analyses were performed using *t* test and one-way ANOVA, with p < 0.05 considered statistically significant.

## Supplementary Material

Supplementary figures and table.

## Abbreviations

ATV: Atorvastatin
Bax: Bcl2 Associated X
Cav1: Caveolin 1
GLUT: Glucose Transporter
TKI: Tyrosine Kinase Inhibitor
MVA: Mevalonic Acid
EGFR: Epidermal Growth Factor Receptor
NSCLC: Non-Small Cell Lung Cancer
FLOT1: Flotillin 1
NADPH: Nicotinamide Adenine Dinucleotide Phosphate
AMPK: 5' adenosine monophosphate-activated protein kinase
NSG: Non-Obese Diabetic Severe Combined Immunodeficiency
CCSP-rTTA: Clara Cell Secretory Protein reverse Tetracycline-controlled Transactivator
PLA: Proximity Ligation Assay
