# Supplementary material for: CAV1 - GLUT3 signaling is important for cellular energy and can be targeted by Atorvastatin in Non-Small Cell Lung Cancer
Source: Theranostics. 2019 Aug 14;9(21):6157–74. doi: 10.7150/thno.35805 (PMC6735519; doi:10.7150/thno.35805)
Supplement: Supplementary file 1 — Supplementary figures and table. [file thnov09p6157s1.pdf]

CAV1 - GLUT3 signaling is important for cellular energy and can be targeted by Atorvastatin in Non-Small Cell Lung Cancer

A Ali et al.

Supplementary table and figures

Cell line	Type	EGFR status	Gefitinib sensitivity	Erlotinib sensitivity
PC-9	NSCLC	delE746-A750	Yes	Yes
PC-9GR	NSCLC	delE746-A750	No	No
H1975	NSCLC	L858R, T790M	No	No
H1703	NSCLC	Wild-type	No	No
NL20	Immortalized non transformed	Wild-type	No	No
HCC4006	NSCLC	delL747-E749,A750P	Yes	Yes
HCC827	NSCLC	delE746-A750	Yes	Yes

Table S1. EGFR mutation status and response to EGFR-TKIs, Gefitinib and Erlotinib, of lung cell lines used in this study.

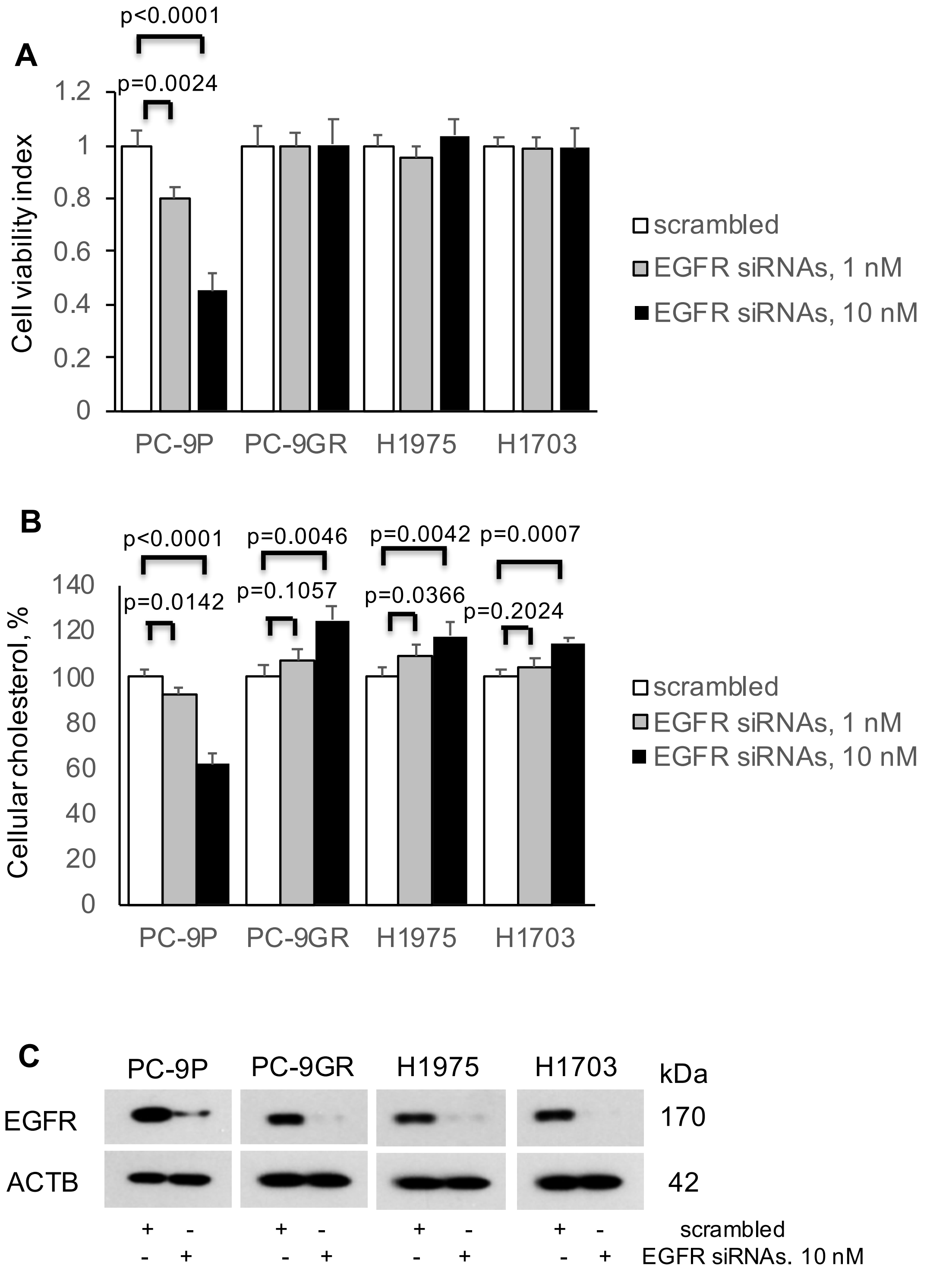

Figure S1. The effects of EGFR knockdown by siRNAs on **A.** cell viability and **B.** cellular cholesterol levels in NSCLC cells. Significance in differences in cell viability and cholesterol levels, in which scrambled acted as control, was determined by t-test. **C.** Western blotting show the efficacy of EGFR knockdown with siRNAs at 10 nM for 72 h.

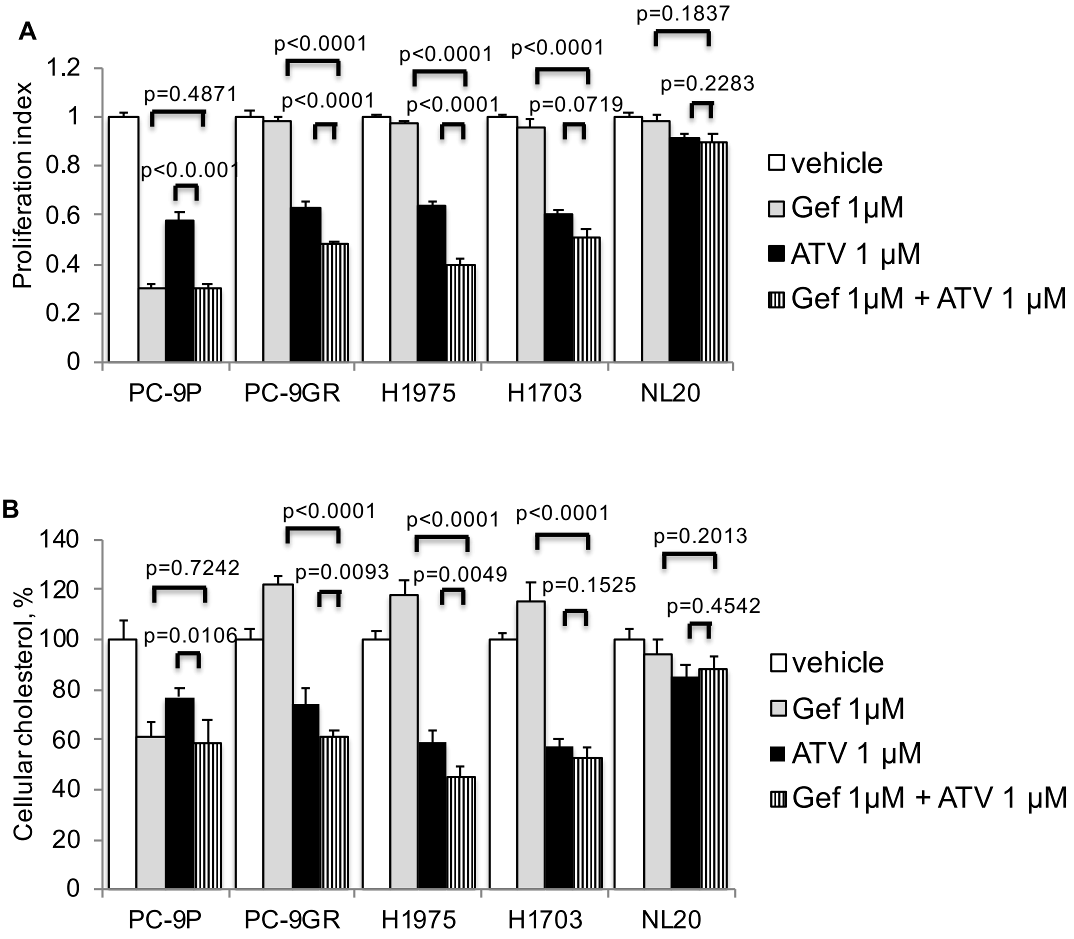

Figure S2. The impact of combination ATV and Gefitinib treatment versus single agent on **A.** cell proliferation and **B.** cellular cholesterol levels in NSCLC and NL20 cells. Significance in differences in cell viability and cholesterol levels, in which Gefitinib or ATV acted as controls, was determined by t test.

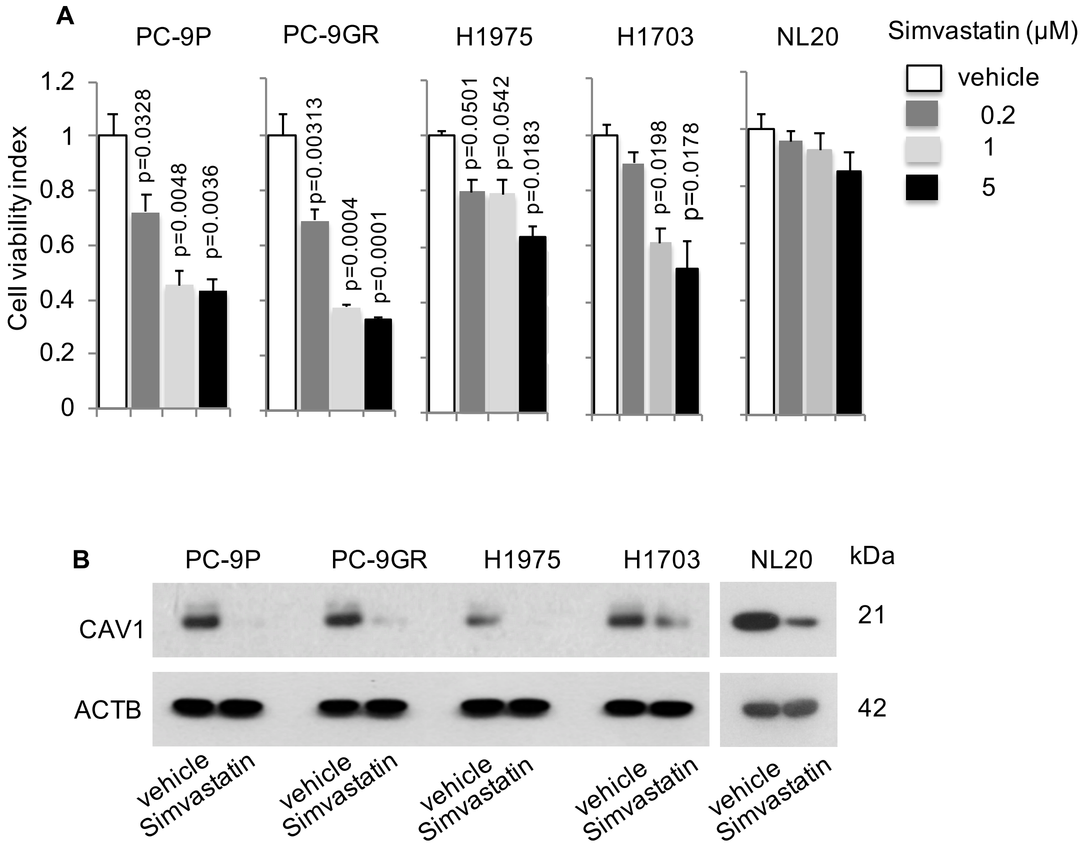

Figure S3. Effects of Simvastatin on NSCLC cells. **(A)** Cell viability assay shows the anti-tumor effects of Simvastatin at the indicated doses in NSCLC cells after 72 h exposure. Significance in differences in cell viability indexes, in which vehicle acted as control, was determined by t test**. (B)** Western blot analyses showing Cav1, GLUT3 and Bax protein levels in vehicle- or Simvastatin-treated (at 1 µM) cells.

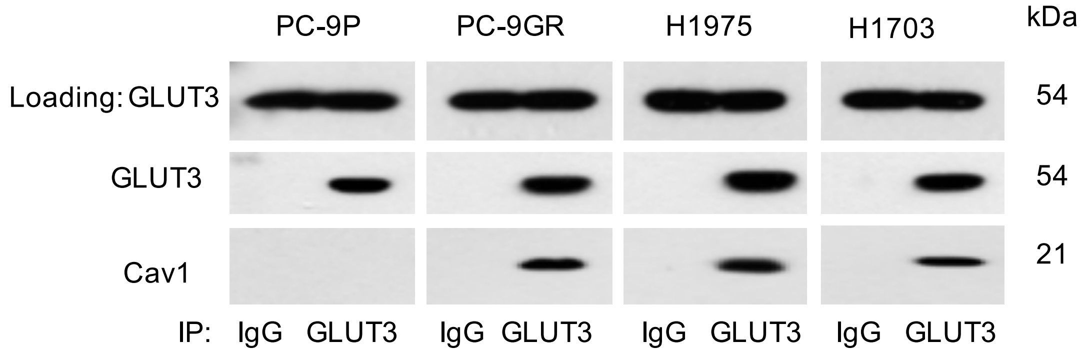

Figure S4. GLUT3 immunoprecipitations from tumor cell extracts and levels of GLUT1 and GLUT3 was determined with respective antibodies. Protein size is indicated in kDa.

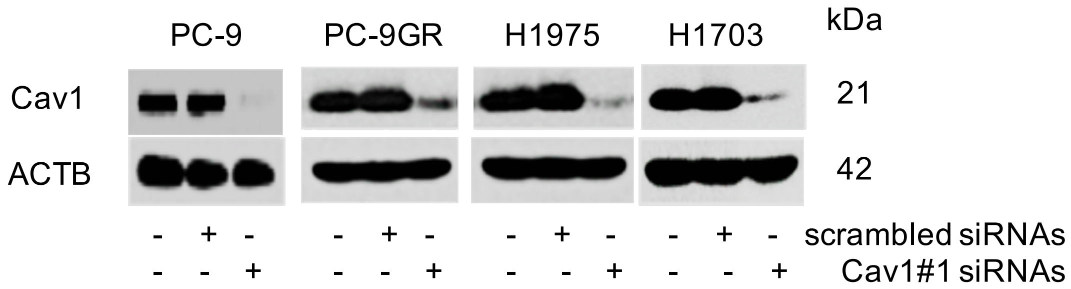

Figure S5. Western blots showing Cav1 knockdown, by siRNAs at 25 nM for 72 h, in NSCLC cells. Protein size is indicated in kDa.

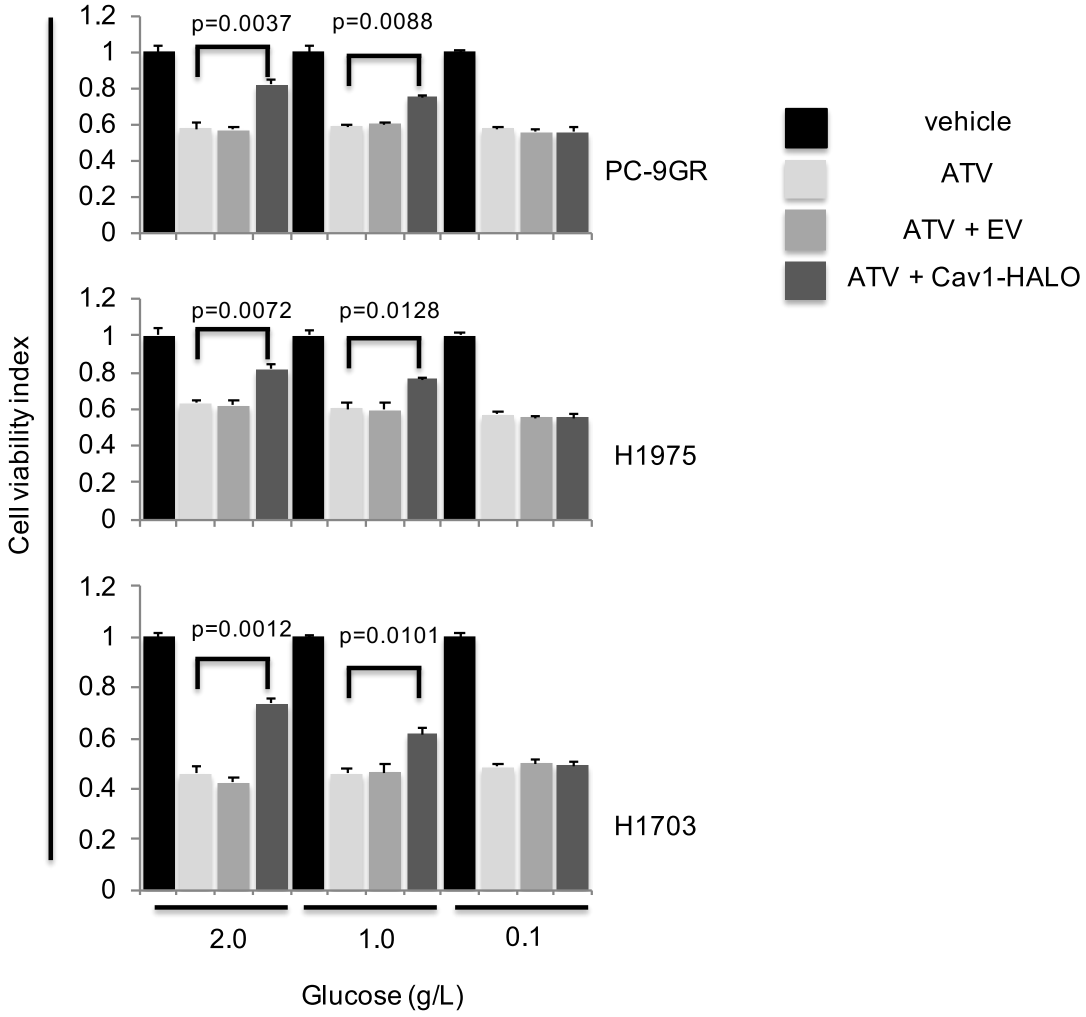

Figure S6. The effect of exogenous glucose on viability of ATV- treated or Cav1-HALO expressiing ATV-treated (at 1 μM) NSCLC cells (n=3). Significance in differences in cell viability indices, in which ATV-treated cells acted as control, was determined by t test.

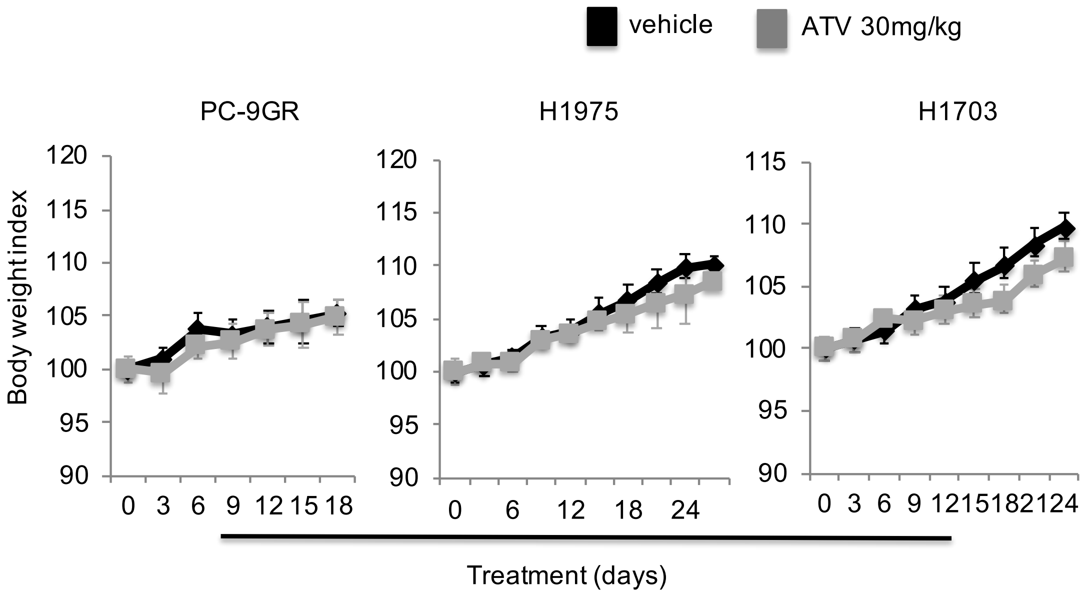

Figure S7. Comparison of body weight between vehicle- and ATV-treated mice carrying xenografts of PC-9GR, H1975 and H1703 NSCLC cells. ATV – 30 mg/kg.

Sample	Luminescence Ratio B/A	Conclusion
Negative control	0.195	negative
Positive control	92.42	positive
PC-9	0.297	negative
PC-9GR	0.422	negative
H1975	0.223	negative
H1703	0.169	negative
NL20	0.141	negative
HCC4006	0.164	negative
HCC827	0.378	negative

Intepretation of:		
Ratio of B/A	<1	negative
	1-1.2	borderline
	>1.2	positive

Figure S8. Results and Interpretation of MycoAlertT assay results on lung cells used in this study.

Centre for Translational Research and Diagnostics
Centre for Translational Medicine Level 11
National University of Singapore
14 Medical Drive
Singapore 117599
Office: +65 65168055, Lab: +65 65165043, Fax: +65 68749664

Cell ID Report

Prepared by:	Sarah Low Hong Hui
Date Prepared:	16 October 2013

1 Sample Information

Run Loading Number	20
Sample ID	Negative
Additional ID Comments	-
Pellet/DNA	-
Estimated Cells in Pellet	-
Received by	-
Date Pellet Received	-
Date DNA Received	-
DNA vol. (µl)	-
DNA conc. (ng/µl)	-
DNA quality (A260/280)	-

DNA Extraction: DNeasy Blood and Tissue Kit (Qiagen, Hilden, Germany)
DNA Analysis: Nanodrop ND1000 (Wilmington, DE)

2 Run Information

Volume DNA Used (µl)	-
Amount DNA Used (ng)	-
Run Date	9th October 2013
Run File Name	Kong_20131009

Cell ID Analysis: Cell ID system (Promega, Madison, WI)
Capillary Electrophoresis: ABI 3130xl Genetic Analyser (Life Technologies, Foster City, CA)

3 Run Results

Locus	Positive	Negative
TH01	6,9.3	-
D21S11	29,31.2	-
D5S818	12	-
D13S317	9,11	-
D7S820	8,11	-
D16S539	9,13	-
CSF1PO	12	-
Amelogenin	X,Y	-
vWA	16,19	-
TPOX	11	-
Reference	2800M control DNA	Negative
Reference Source	Promega	ATCC
Percent Match	100%	100%
Status	Passed	Passed

Analysis: Gene mapper V4.0 software. (Life Technologies, Foster City, CA)
Numbers indicate the allele designations for each locus.
Electrophoragram is added below under Appendix for your reference
Percent Match according to ICLAC Match Criteria Worksheet v1.1 (http://standards.atcc.org)

4 References

ANSI/ATCC ASN-0002-2011. Authentication of Human Cell Lines: Standardization of STR Profiling. ANSI eStandards Store, 2012.

Centre for Translational Medicine Level 11
National University of Singapore
14 Medical Drive
Singapore 117599
Office: +65 65168055, Lab: +65 65165043, Fax: +65 68749664

Cell ID Report

Prepared by:	Sarah Low Hong Hui
Date Prepared:	16 October 2013

1. Sample Information

Run Loading Number	18
Sample ID	PC-9
Additional ID Comments	-
Pellet/DNA	-
Estimated Cells in Pellet	-
Received by	-
Date Pellet Received	-
Date DNA Received	-
DNA vol. (µl)	-
DNA conc. (ng/µl)	-
DNA quality (A260/280)	-

DNA Extraction: DNeasy Blood and Tissue Kit (Qiagen, Hilden, Germany)
DNA Analysis: Nanodrop ND1000 (Wilmington, DE)

2. Run Information

Volume DNA Used (µl)	-
Amount DNA Used (ng)	-
Run Date	9th October 2013
Run File Name	Kong_20131009

Cell ID Analysis: Cell ID system (Promega, Madison, WI)
Capillary Electrophoresis: ABI 3130xl Genetic Analyser (Life Technologies, Foster City, CA)

3. Run Results

Locus	Positive	PC-9
TH01	6,9.3	7
D21S11	29,31.2	30
D5S818	12	11
D13S317	9,11	8
D7S820	8,11	10,11
D16S539	9,13	9
CSF1PO	12	11
Amelogenin	X,Y	X

vWA	16,19	17
TPOX	11	11
Reference	2800M control DNA	PC-9
Reference Source	Promega	DSMZ
Percent Match	100%	100%
Status	Passed	Passed

Analysis: Gene mapper V4.0 software. (Life Technologies, Foster City, CA)
Numbers indicate the allele designations for each locus.
Electrophoragram is added below under Appendix for your reference
Percent Match according to ICLAC Match Criteria Worksheet v1.1 (http://standards.atcc.org)

4. References

ANSI/ATCC ASN-0002-2011. Authentication of Human Cell Lines: Standardization of STR Profiling. ANSI eStandards Store, 2012.

Centre for Translational Medicine Level 11
National University of Singapore
14 Medical Drive
Singapore 117599
Office: +65 65168055, Lab: +65 65165043, Fax: +65 68749664

Cell ID Report

Prepared by:	Sarah Low Hong Hui
Date Prepared:	16 October 2013

1. Sample Information

Run Loading Number	11
Sample ID	H1975
Additional ID Comments	-
Pellet/DNA	-
Estimated Cells in Pellet	-
Received by	-
Date Pellet Received	-
Date DNA Received	-
DNA vol. (µl)	-
DNA conc. (ng/µl)	-
DNA quality (A260/280)	-

DNA Extraction: DNeasy Blood and Tissue Kit (Qiagen, Hilden, Germany)
DNA Analysis: Nanodrop ND1000 (Wilmington, DE)

2. Run Information

Volume DNA Used (µl)	-
Amount DNA Used (ng)	-
Run Date	9th October 2013
Run File Name	Kong_20131009

Cell ID Analysis: Cell ID system (Promega, Madison, WI)
Capillary Electrophoresis: ABI 3130xl Genetic Analyser (Life Technologies, Foster City, CA)

3. Run Results

Locus	Positive	H1975
TH01	6,9.3	7
D21S11	29,31.2	28
D5S818	12	11,12
D13S317	9,11	10,13
D7S820	8,11	8
D16S539	9,13	9,12
CSF1PO	12	12
Amelogenin	X,Y	X

vWA	16,19	18
TPOX	11	8,11
Reference	2800M control DNA	NCI- H1975
Reference Source	Promega	ATCC
Percent Match	100%	96.4%
Status	Passed	Passed

Analysis: Gene mapper V4.0 software. (Life Technologies, Foster City, CA)
Numbers indicate the allele designations for each locus.
Electrophoragram is added below under Appendix for your reference
Percent Match according to ICLAC Match Criteria Worksheet v1.1 (http://standards.atcc.org)

4. References

ANSI/ATCC ASN-0002-2011. Authentication of Human Cell Lines: Standardization of STR Profiling. ANSI eStandards Store, 2012.

Centre for Translational Medicine Level 11
National University of Singapore
14 Medical Drive
Singapore 117599
Office: +65 65168055, Lab: +65 65165043, Fax: +65 68749664

Cell ID Report

Prepared by:	Sarah Low Hong Hui
Date Prepared:	16 October 2013

1. Sample Information

Run Loading Number	10
Sample ID	H1703
Additional ID Comments	-
Pellet/DNA	-
Estimated Cells in Pellet	-
Received by	-
Date Pellet Received	-
Date DNA Received	-
DNA vol. (µl)	-
DNA conc. (ng/µl)	-
DNA quality (A260/280)	-

DNA Extraction: DNeasy Blood and Tissue Kit (Qiagen, Hilden, Germany)
DNA Analysis: Nanodrop ND1000 (Wilmington, DE)

2. Run Information

Volume DNA Used (µl)	-
Amount DNA Used (ng)	-
Run Date	9th October 2013
Run File Name	Kong_20131009

Cell ID Analysis: Cell ID system (Promega, Madison, WI)
Capillary Electrophoresis: ABI 3130xl Genetic Analyser (Life Technologies, Foster City, CA)

3. Run Results

Locus	Positive	H1703
TH01	6,9.3	7
D21S11	29,31.2	29
D5S818	12	11
D13S317	9,11	10
D7S820	8,11	10,12
D16S539	9,13	10,12
CSF1PO	12	12
Amelogenin	X,Y	X,Y

vWA	16,19	16,17
TPOX	11	8,11
Reference	2800M control DNA	NCI- H1703
Reference Source	Promega	ATCC
Percent Match	100%	100%
Status	Passed	Passed

Analysis: Gene mapper V4.0 software. (Life Technologies, Foster City, CA)
Numbers indicate the allele designations for each locus.
Electrophoragram is added below under Appendix for your reference
Percent Match according to ICLAC Match Criteria Worksheet v1.1 (http://standards.atcc.org)

4. References

ANSI/ATCC ASN-0002-2011. Authentication of Human Cell Lines: Standardization of STR Profiling. ANSI eStandards Store, 2012.

Centre for Translational Medicine Level 11
National University of Singapore
14 Medical Drive
Singapore 117599
Office: +65 65168055, Lab: +65 65165043, Fax: +65 68749664

CTRAD

Cell ID Report

Prepared by:	Sarah Low Hong Hui
Date Prepared:	16 October 2013

1. Sample Information

Run Loading Number	16
Sample ID	HCC827
Additional ID Comments	-
Pellet/DNA	-
Estimated Cells in Pellet	-
Received by	-
Date Pellet Received	-
Date DNA Received	-
DNA vol. (µl)	-
DNA conc. (ng/µl)	-
DNA quality (A260/280)	-

DNA Extraction: DNeasy Blood and Tissue Kit (Qiagen, Hilden, Germany)
DNA Analysis: Nanodrop ND1000 (Wilmington, DE)

2. **Run Information**

Volume DNA Used (µl)	-
Amount DNA Used (ng)	-
Run Date	9th October 2013
Run File Name	Kong_20131009

Cell ID Analysis: Cell ID system (Promega, Madison, WI)
Capillary Electrophoresis: ABI 3130xl Genetic Analyser (Life Technologies, Foster City, CA)

3. **Run Results**

**Locus**	**Positive**	**HCC827**
TH01	6,9.3	6
D21S11	29,31.2	31
D5S818	12	12
D13S317	9,11	9
D7S820	8,11	11,12
D16S539	9,13	12
CSF1PO	12	11
Amelogenin	X,Y	X

vWA	16,19	18
TPOX	11	8
Reference	2800M control DNA	HCC827
Reference Source	Promega	ATCC
Percent Match	100%	100%
Status	Passed	Passed

Analysis: Gene mapper V4.0 software. (Life Technologies, Foster City, CA)
Numbers indicate the allele designations for each locus.
Electrophoragram is added below under Appendix for your reference
Percent Match according to ICLAC Match Criteria Worksheet v1.1 (http://standards.atcc.org)

4. References

ANSI/ATCC ASN-0002-2011. Authentication of Human Cell Lines: Standardization of STR Profiling. ANSI eStandards Store, 2012.

Cell Line Authentication Service
STR Profile Report

Sample Submitted By: Teo Jun Ting

Email Address: teo.jun.ting83@gmail.com

Order ID: AITB-CLA 001

Cell Line Designation: CRL-2871

Date Sample Received: 4th Oct 2017 **Report Date:** 13th Oct 2017

Methodology: Ten short tandem repeat (STR) loci plus the gender determining locus, Amelogenin, were amplified using the commercially available GenePrint ® 10 System from Promega. The cell line sample was processed using the ABI Prism® 3730xl Genetic Analyzer. Data were analyzed using GeneMapper® 4.1 software (Applied Biosystems). Appropriate positive and negative controls were run and confirmed for each sample submitted. Known reference profiling against the ATCC STR database with the STR profile for your human cell line.

Data Interpretation: Cell lines were authenticated using Short Tandem Repeat (STR) analysis as described in 2012 in ANSI Standard (ASN-0002) by the ATCC Standards Development Organization (SDO) and in Capes-Davis et al., Match criteria for human cell line authentication: Where do we draw the line? Int. J. Cancer. 2012 Nov 8. doi: 0.1002/ijc.27931

Test Results for Submitted Sample					ATCC Reference Database Profile			
**Loci**	**Query Profile: HCC4006**				**Database Profile: CRL-2871**			
**TH01**	7				7			
**D21S11**	31							
**D5S818**	12				12			
**D13S317**	11	12			11	12		
**D7S820**	9	12			9	12		
**D16S539**	11	12			11	12		
**CSF1PO**	10				10			
**Amelogenin**	X				X			
**Vwa**	16	17			16	17		
**TPOX**	8	9			8	9		
Number of shared alleles between query sample and database profile:								14
Total number of alleles in the database profile:								14
Percent match between in the submitted sample and the database profile:								100
The alleles match algorithm compares the 8 core loci plus amelogenin only, even though alleles from all loci will be reported when available.								
**NOTE:** Loci highlighted in grey (8 core STR loci plus Amelogenin) can be made public to verify cell identity. In order to protect the identity of the donor, please do not publish the allele calls from the STR loci tested. Electropherograms showing raw data are attached.								

Explanation of Test Results

Cell lines with ≥ 80% match are considered to be related; i.e., derived from a common ancestry.

Cell lines with between a 55% to 80% match require further profiling for authentication of relatedness.

☐ The submitted sample profile is human, but not a match for any profile in the ATCC STR database

√ The submitted sample is an exact match for the following ATCC human cell line(s) in the ATCC STR database (8 core loci plus Amelogenin): CRL-2871

☐ The submitted sample is similar to the following ATCC human cell line(s):

**Performed By Lab Technician:**	Jennifer Ng 13/10/2017
**Reviewed By:**	Sathya 13/10/2017

Figure S9. Authentication of cell lines used in this study.
